# Supplementary material for: Early decarbonisation of the European energy system pays off
Source: Nat Commun. 2020 Dec 4;11:6223. doi: 10.1038/s41467-020-20015-4 (PMC7718908; doi:10.1038/s41467-020-20015-4)
Supplement: Supplementary file 1 — Supplementary Information [file 41467_2020_20015_MOESM1_ESM.pdf]

Supplementary Materials for "Early decarbonisation of the European energy system pays off"

Marta Victoria[a,b,*], Kun Zhu[a], Tom Brown[c], Gorm B. Andresen[a,b], Martin Greiner[a,b]

[a] *Department of Engineering, Aarhus University, Inge Lehmanns Gade 10, 8000 Aarhus, Denmark*
[b] *iCLIMATE Interdisciplinary Centre for Climate Change, Aarhus University*
[c] *Institute for Automation and Applied Informatics (IAI), Karlsruhe Institute of Technology (KIT), Forschungszentrum 449, 76344, Eggenstein-Leopoldshafen, Germany*

*Corresponding author
Email address: mvp@eng.au.dk (Marta Victoria)

Supplementary Figures.

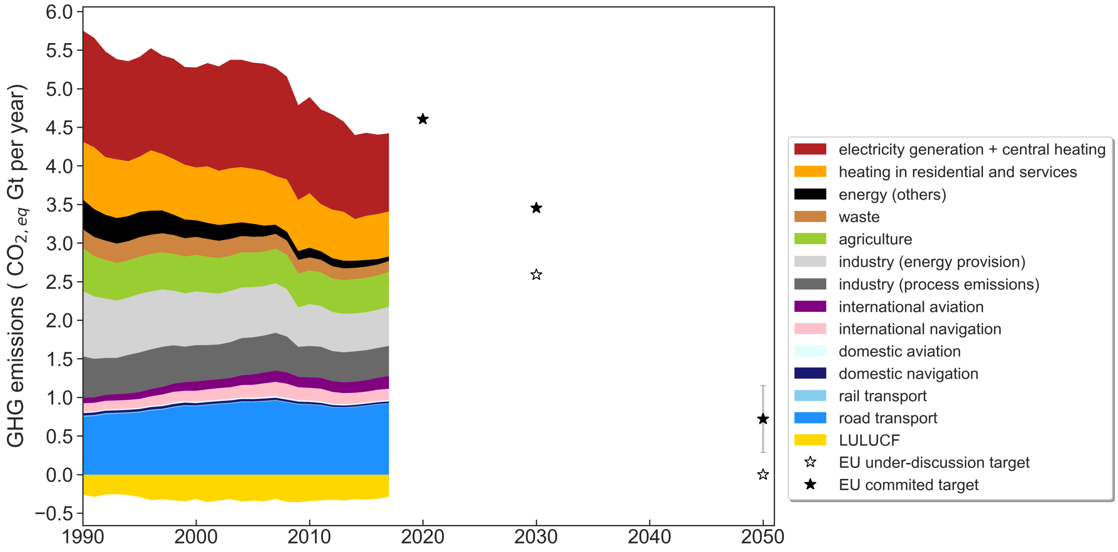

Supplementary Figure 1: Sectoral distribution of historical emissions in the European Union [1]. The black stars indicate committed EU reduction targets, while white stars mark targets under discussion in 2020. LULUCF stands for land use, land-use change, and forestry. In the UNFCCC inventory [1], emissions from electricity and central heating are reported in the same category since they include emissions from Combined Heat and Power (CHP) units that generate electricity and heat that is then used in district heating systems.

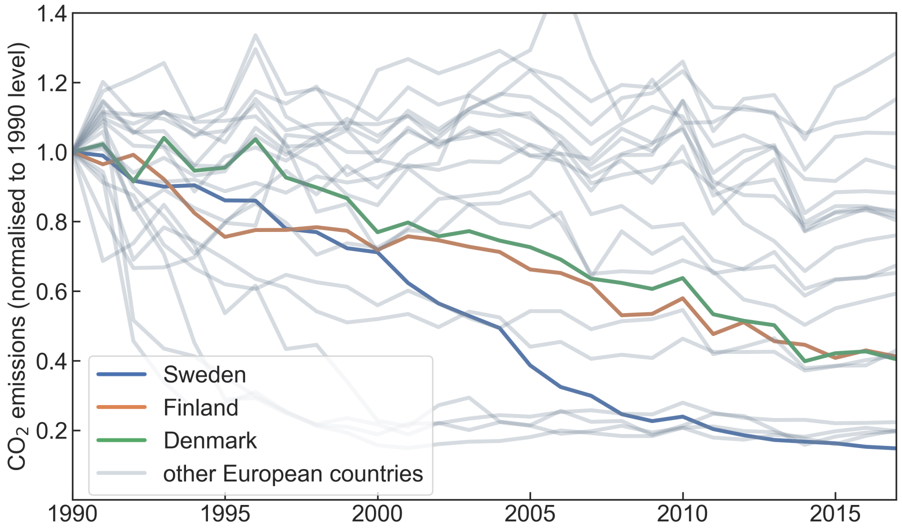

Supplementary Figure 2: Historical CO_2 emissions from the supply of heating in the residential and services sector [1].

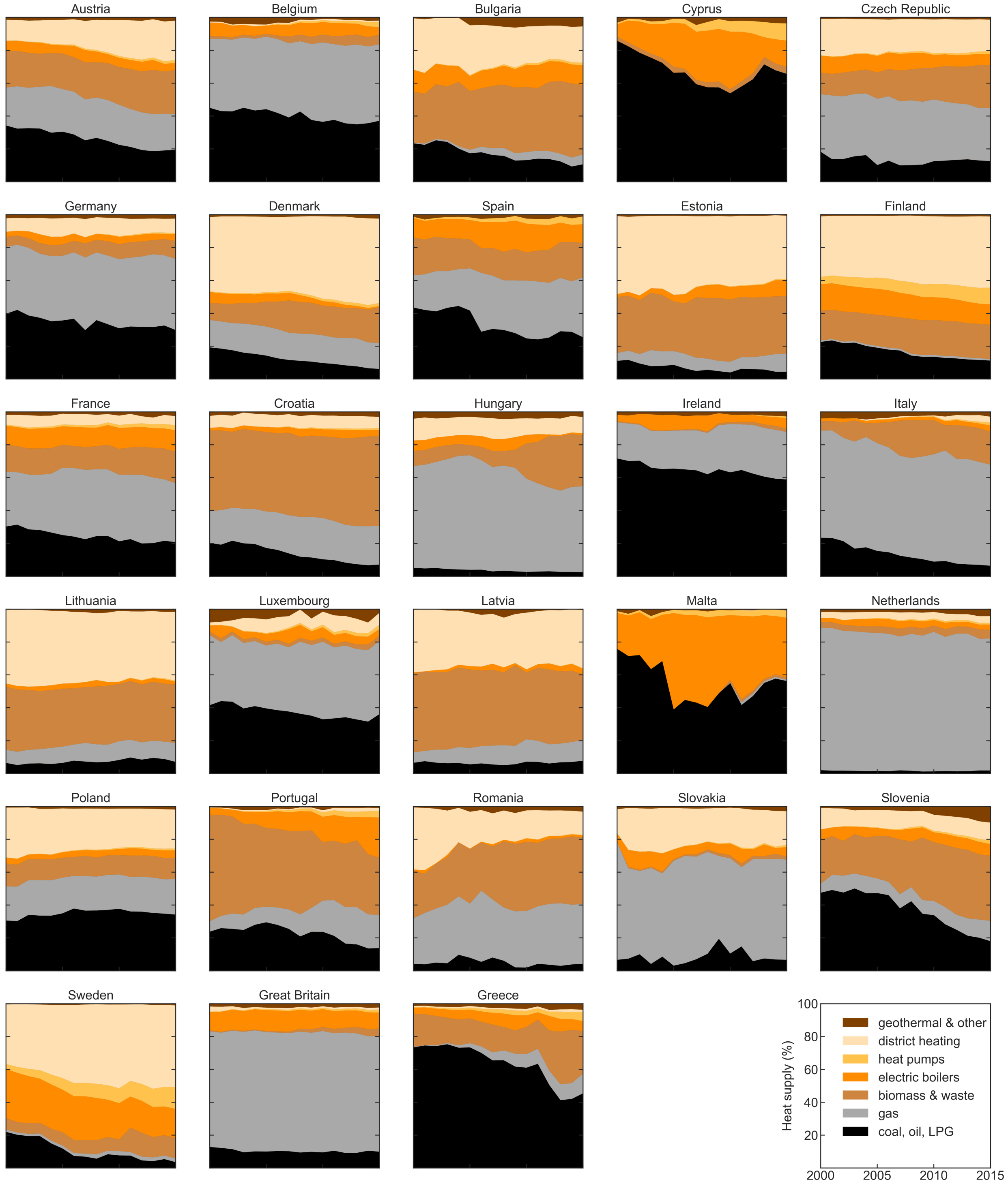

Supplementary Figure 3: Historical share of technologies used to supply heating demand in the residential and services sector [2].

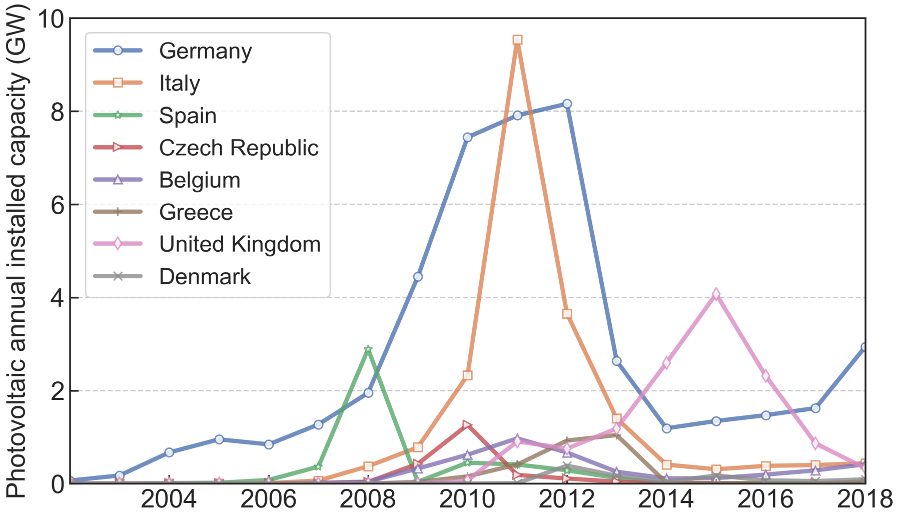

Supplementary Figure 4: Photovoltaic annual build rates for those European countries with a prominent peak [3]. The sharp increases and subsequent decreases in the installation rates were caused by country-specific successive changes in the regulatory frameworks. See for instance [4, 5].

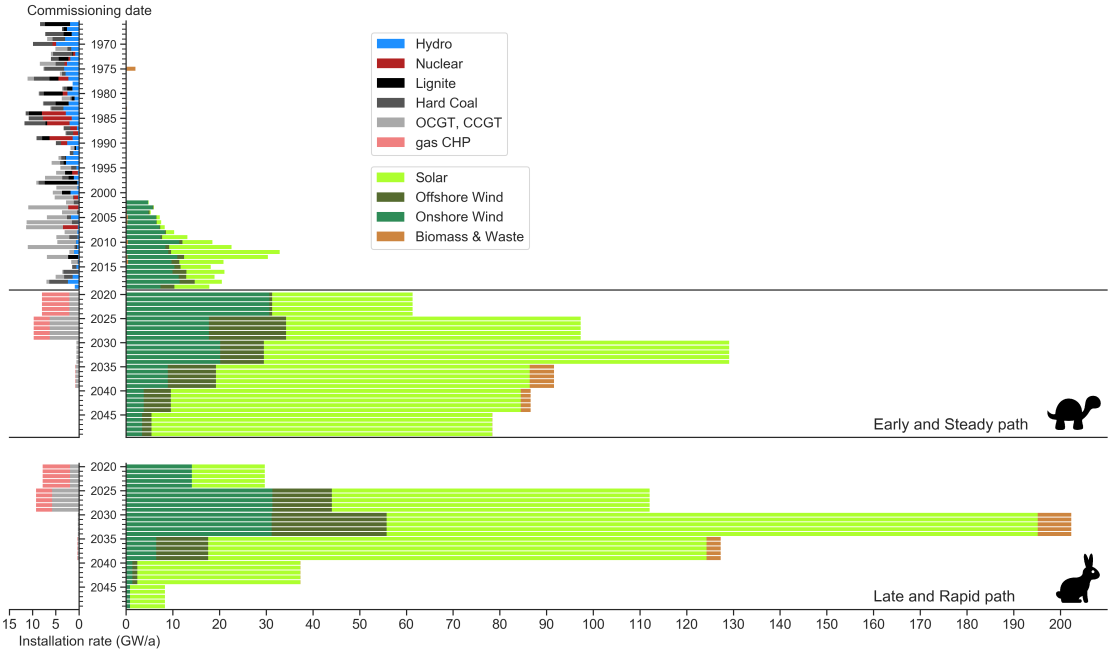

Supplementary Figure 5: Age distribution of European power plants in operation [3, 6] and required annual installation throughout the Early and Steady and Late and Rapid paths.

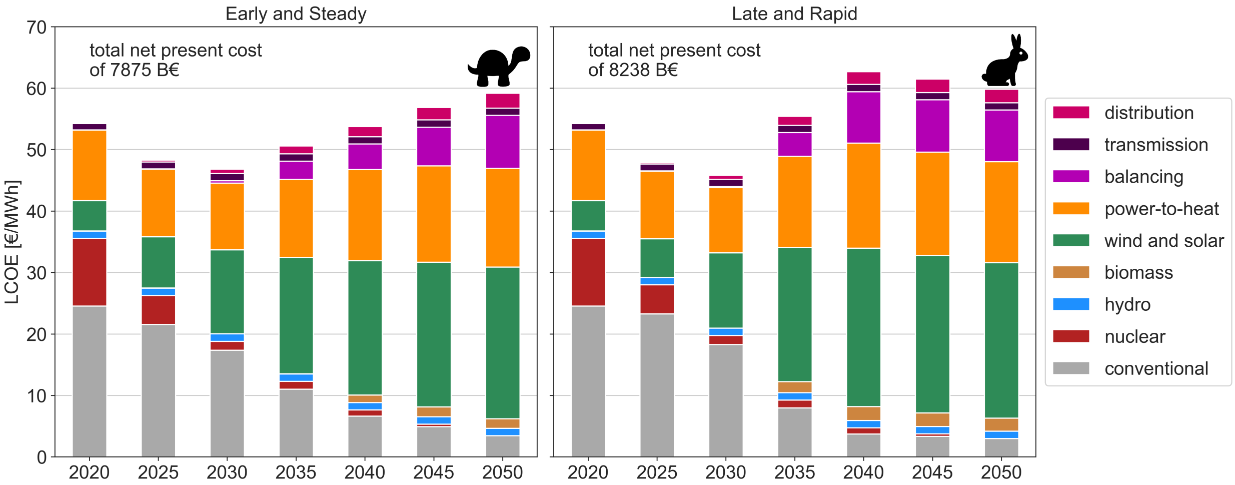

Supplementary Figure 6: Levelized Cost of Energy (LCOE) for the European electricity and heating system throughout transition paths Early and Steady and Late and Rapid shown in Fig. 1 in the main text. Conventional includes costs associated with coal, lignite, and gas power plants producing electricity as well as costs for fossil-fueled boilers and CHP units. Power-to-heat includes costs associated with heat pumps and heat resistors. Balancing includes costs of electric batteries, H_2 storage, and methanation.

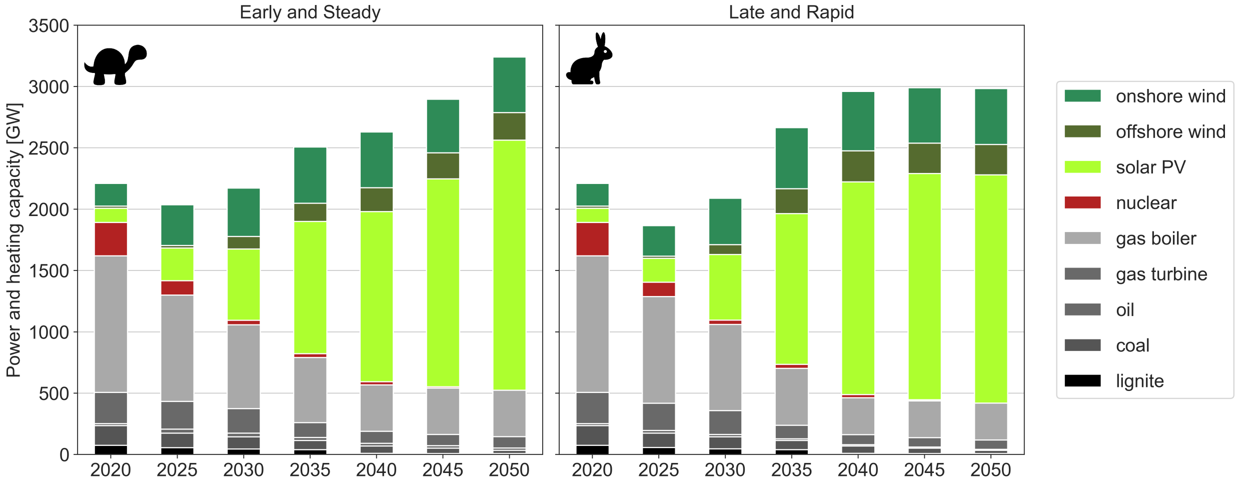

Supplementary Figure 7: Installed capacities for different technologies throughout transition paths shown in Fig. 1 in the main text.

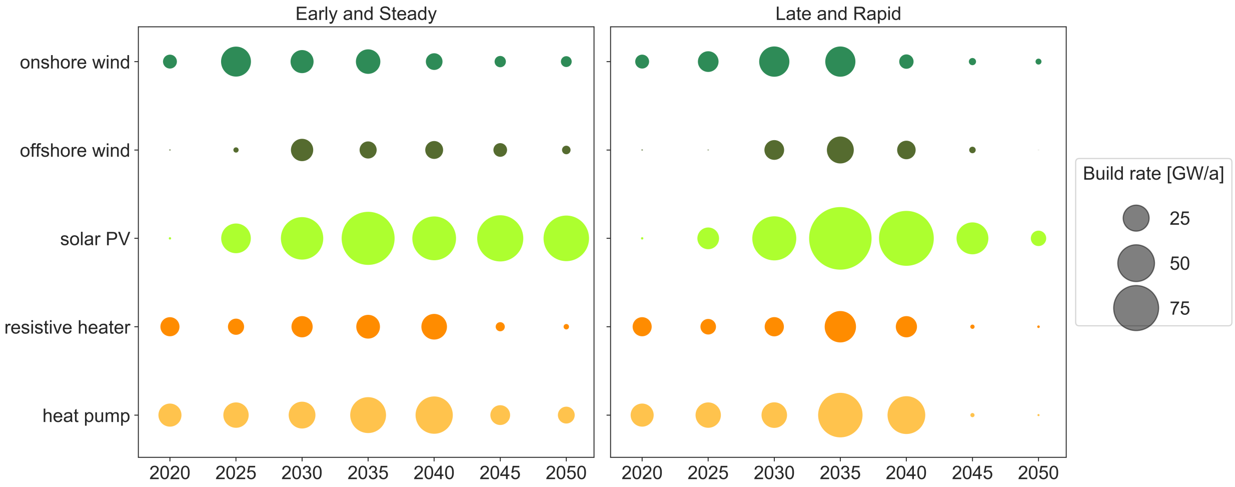

Supplementary Figure 8: Annual build rates for different technologies throughout transition paths shown in Fig. 1 in the main text.

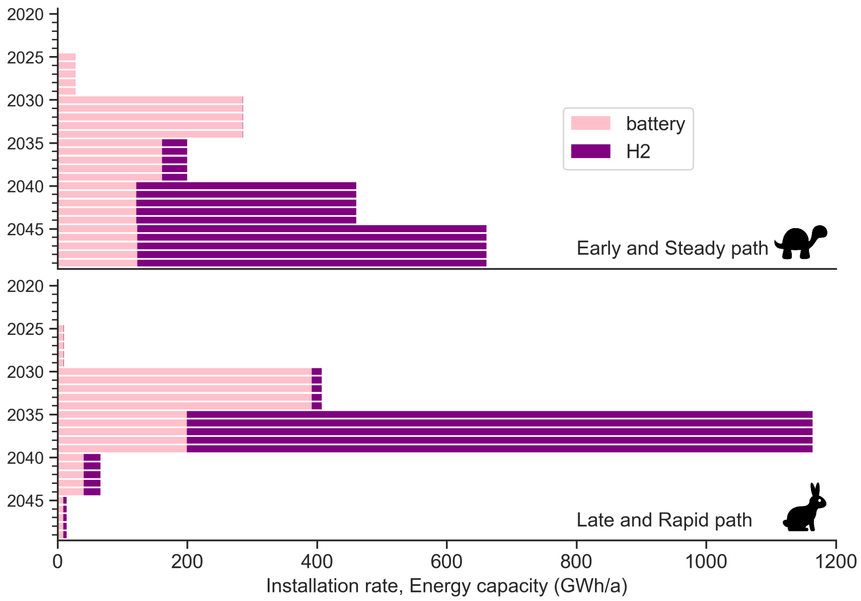

Supplementary Figure 9: Annual build rates for batteries and hydrogen storage throughout transition paths shown in Fig. 1 in the main text.

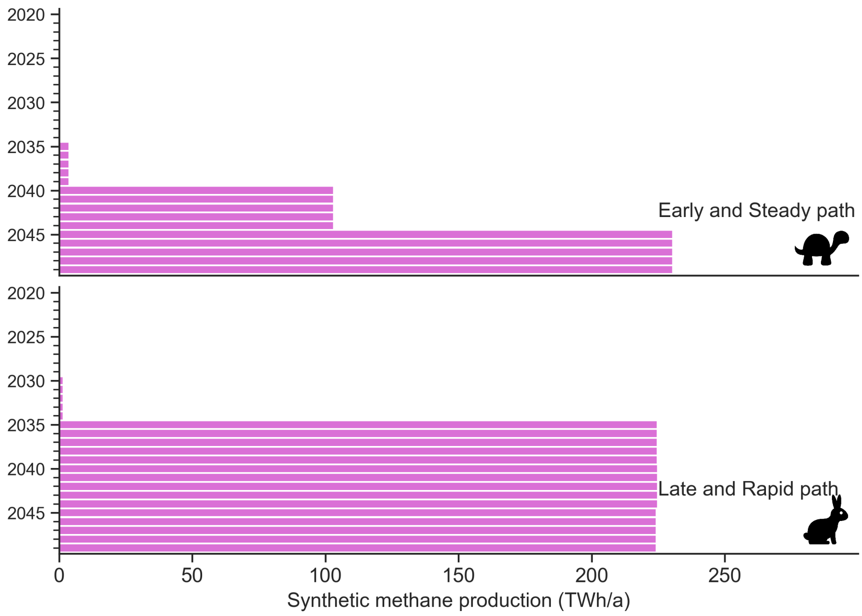

Supplementary Figure 10: Annual synthetic methane production throughout transition paths shown in Fig. 1 in the main text.

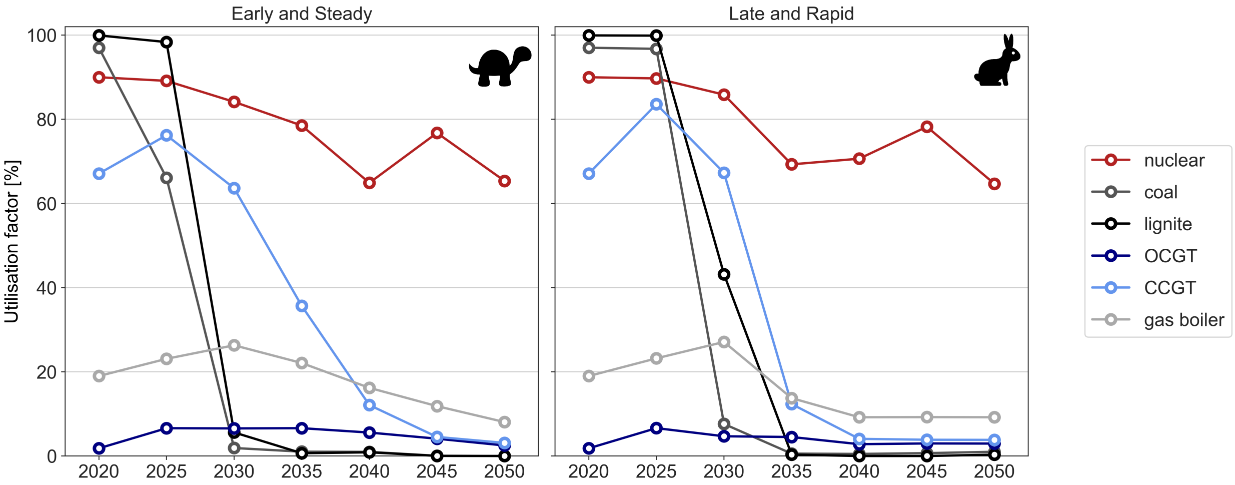

Supplementary Figure 11: Utilisation factors for lignite, coal, OCGT, CCGT, nuclear power plants and gas boilers throughout transition paths shown in Fig. 1 in the main text.

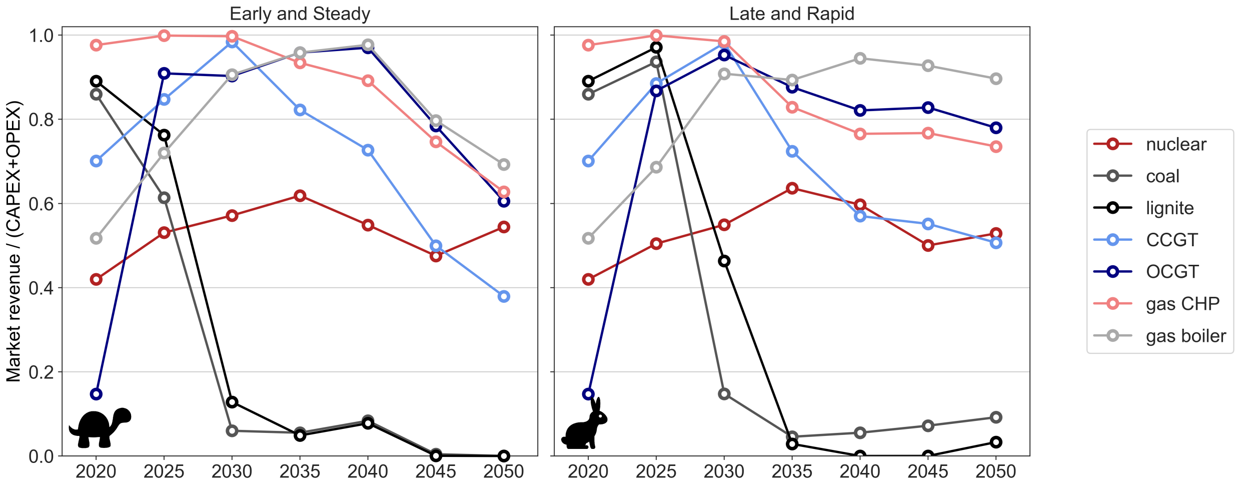

Supplementary Figure 12: Ratio of market revenues to total expenditure for lignite, coal, OCGT, CCGT, nuclear power plants and gas boilers throughout transition paths shown in Fig. 1 in the main text. Total expenditure includes fixed and variable costs, fuel costs and cost associated with CO_2 price.

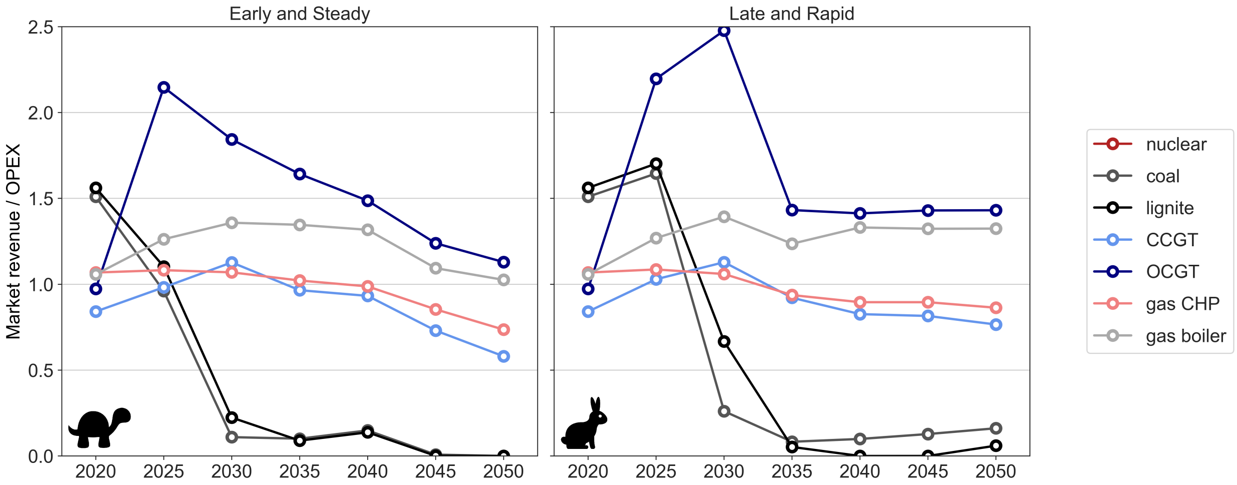

Supplementary Figure 13: Ratio of market revenues to operational expenditure (OPEX) for lignite, coal, OCGT, CCGT, nuclear power plants and gas boilers throughout transition paths shown in Fig. 1 in the main text. OPEX includes fixed and variable operation and maintenance costs, fuel costs and cost associated with CO_2 price.

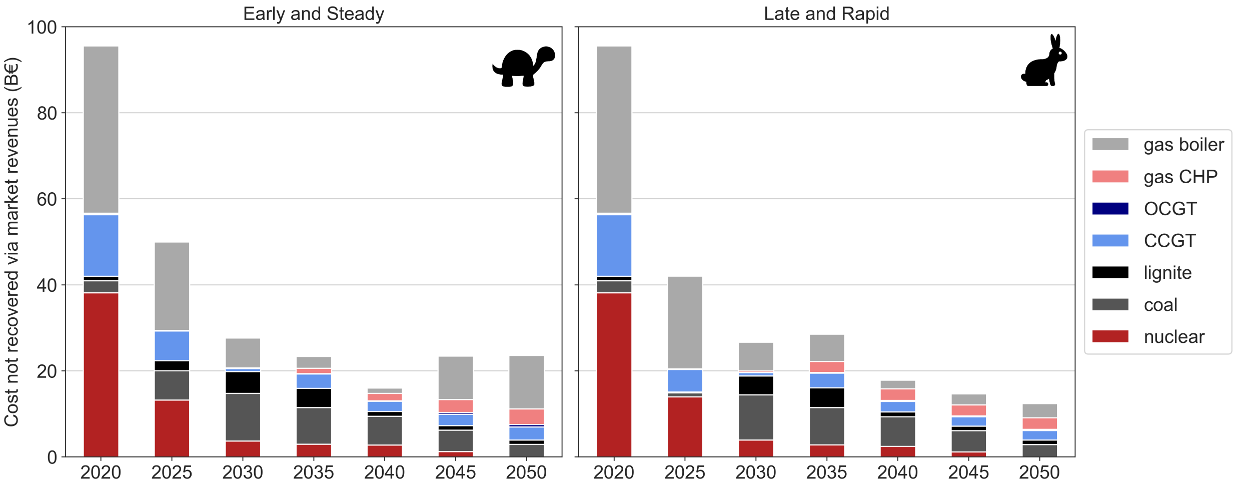

Supplementary Figure 14: Expenditures not recovered via market revenues for lignite, coal, OCGT, CCGT, nuclear power plants and gas boilers throughout transition paths shown in Fig. 1 in the main text.

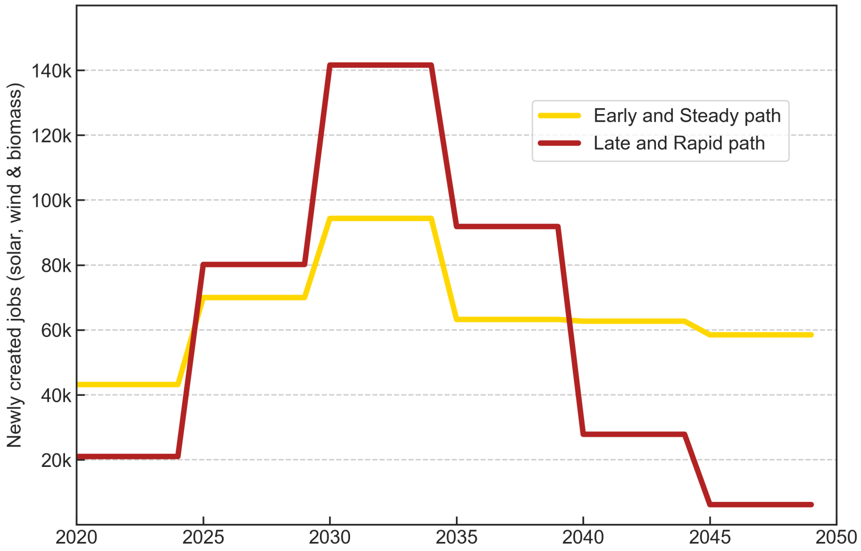

Supplementary Figure 15: Estimated new jobs in wind, solar PV, and biomass throughout transition paths shown in Fig.1 in the main text.

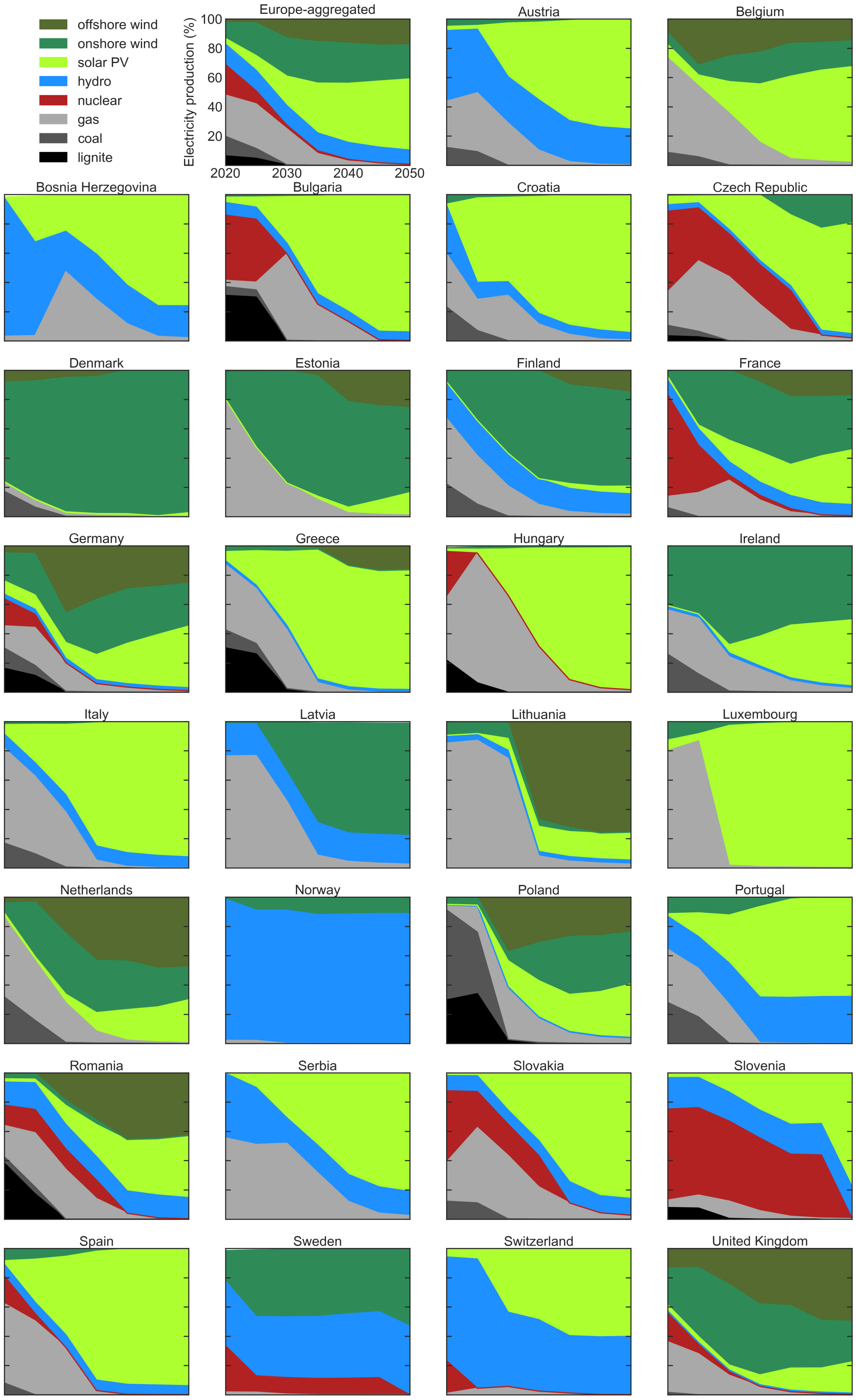

Supplementary Figure 16: Evolution of the electricity generation mix in every country for the Early and Steady transition path. Fuel used in OCGT plants is synthetic methane produced by combining electrolysed-H_2 and direct-air-captured CO_2.

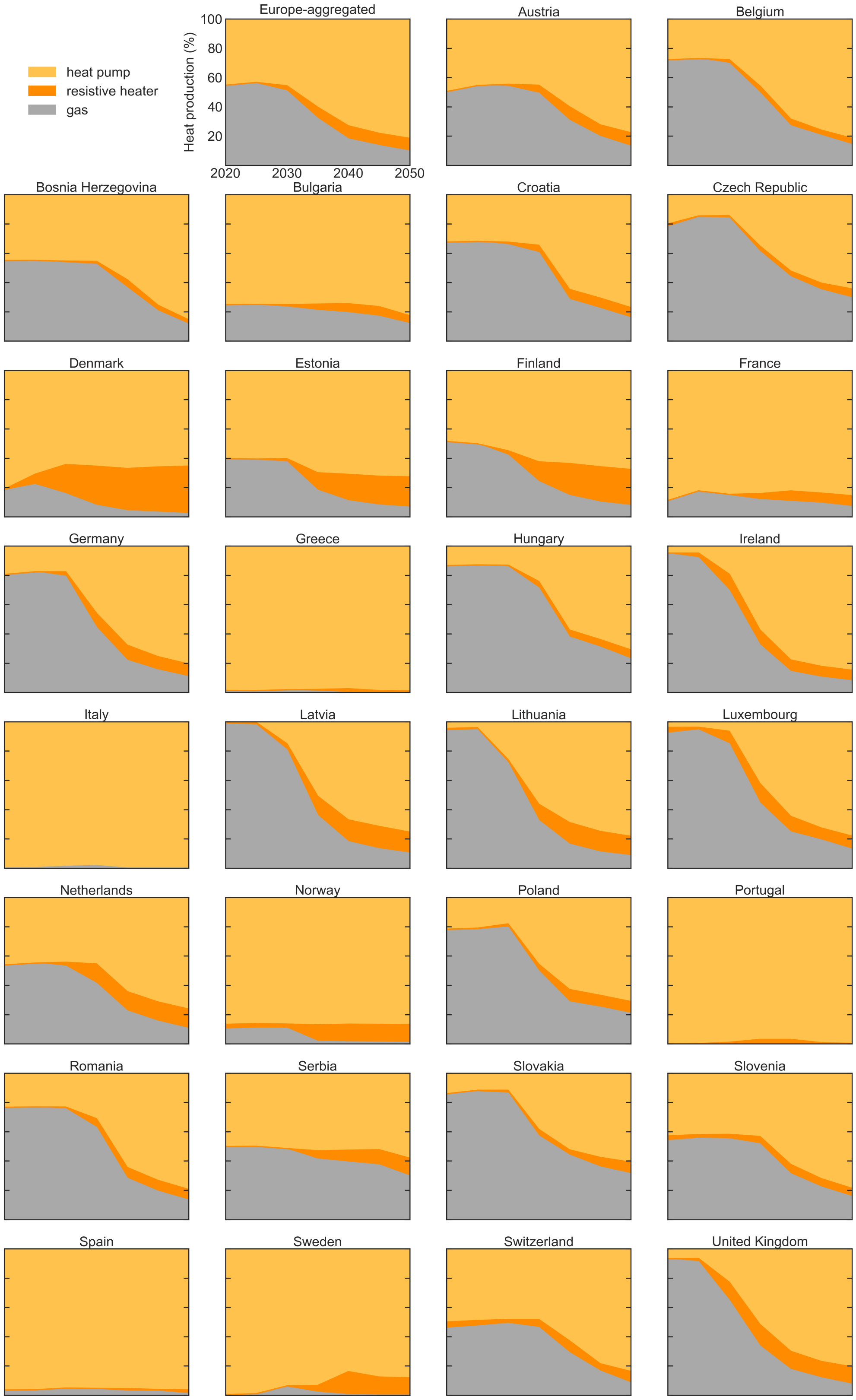

Supplementary Figure 17: Evolution of technologies used to supply heating in the residential and services sector in the Early and Steady path. Fuel used in gas boilers is synthetic methane produced by combining electrolysed-H_2 and direct-air-captured CO_2.

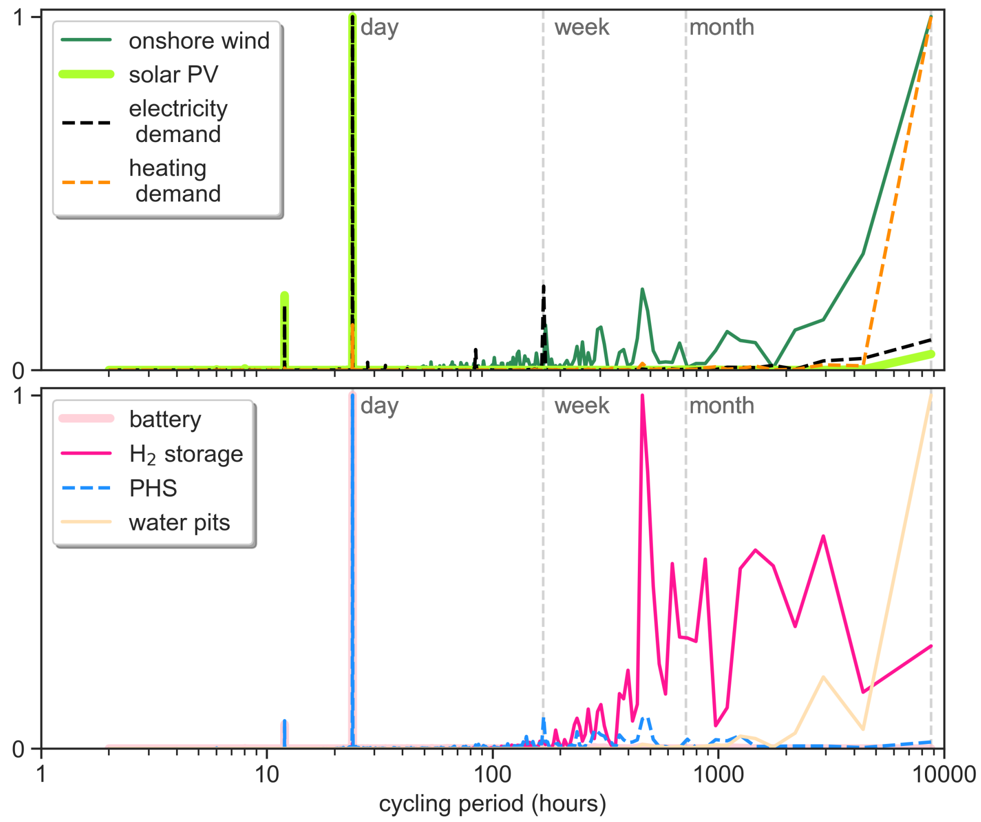

Supplementary Figure 18: Fourier power spectra of wind and solar PV generation, electricity and heating demand, as well as storage technologies dispatch. Time series represent the Europe-aggregated generation/demand for the Early and Steady path in 2050.

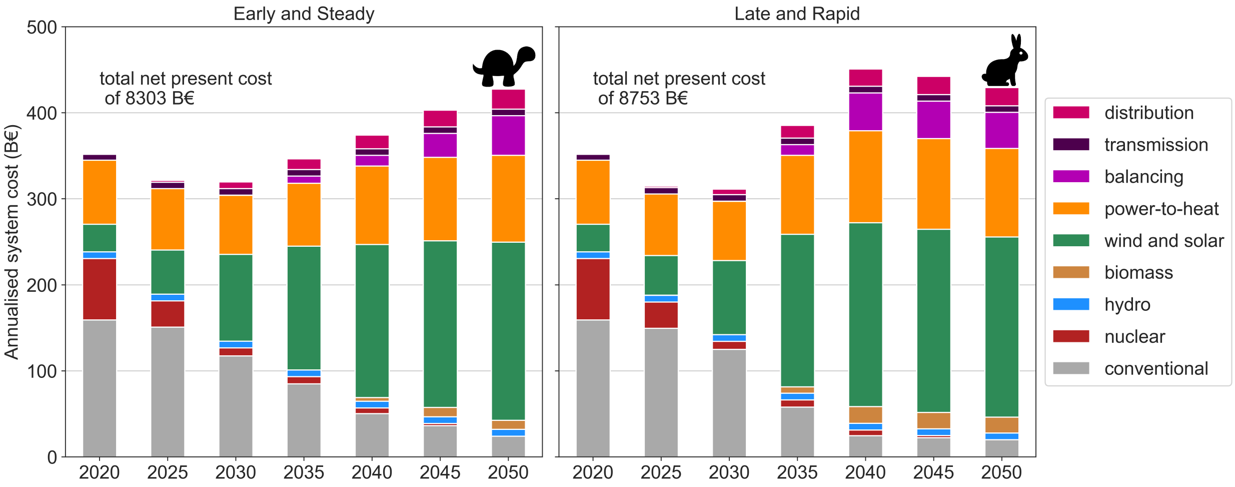

Supplementary Figure 19: Annualised system cost for the European electricity, heating and transport system throughout transition paths Early and Steady and Late and Rapid. Conventional includes costs associated with coal, lignite, and gas power plants producing electricity as well as costs for fossil-fueled boilers and CHP units. Power-to-heat includes costs associated with heat pumps and heat resistors. Balancing includes costs of electric batteries, H_2 storage, and methanation.

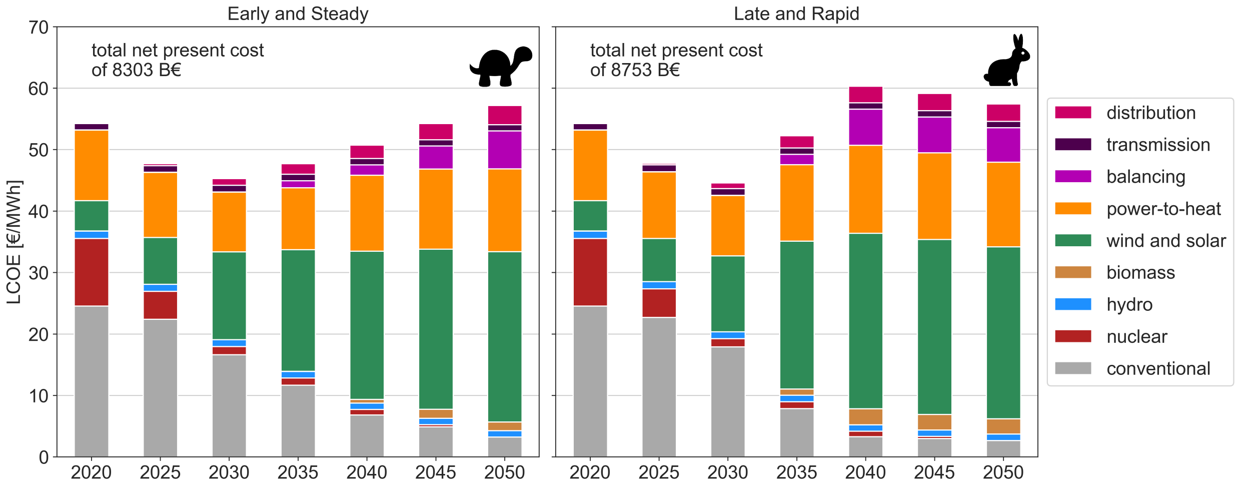

Supplementary Figure 20: Levelized Cost of Energy (LCOE) for the European electricity, heating and transport system throughout transition paths Early and Steady and Late and Rapid. Conventional includes costs associated with coal, lignite, and gas power plants producing electricity as well as costs for fossil-fueled boilers and CHP units. Power-to-heat includes costs associated with heat pumps and heat resistors. Balancing includes costs of electric batteries, H_2 storage, and methanation.

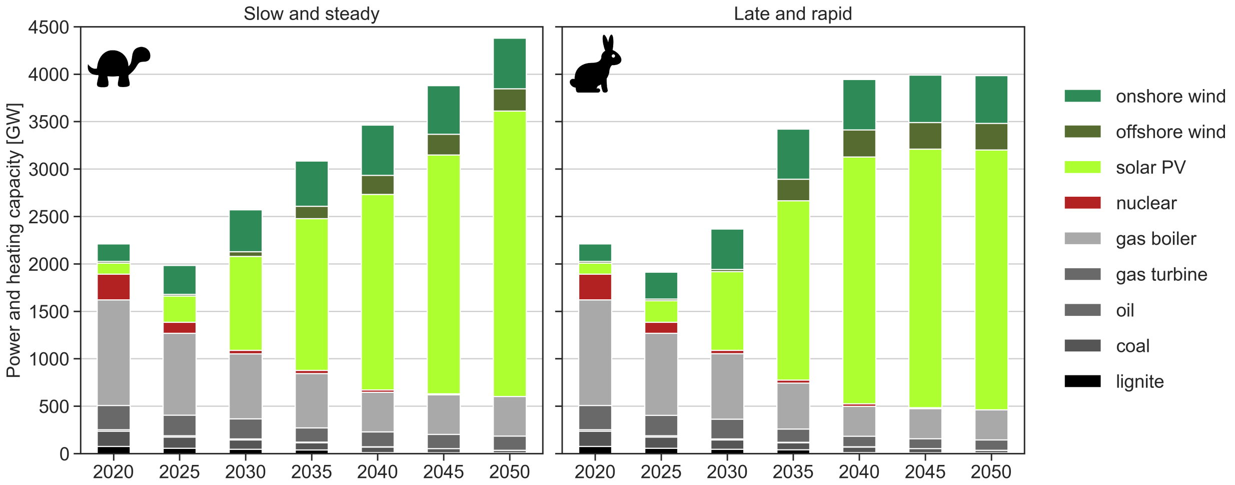

Supplementary Figure 21: Installed capacities for different technologies throughout transition paths Early and Steady and Late and Rapid when the electricity, heating and transport sectors are included.

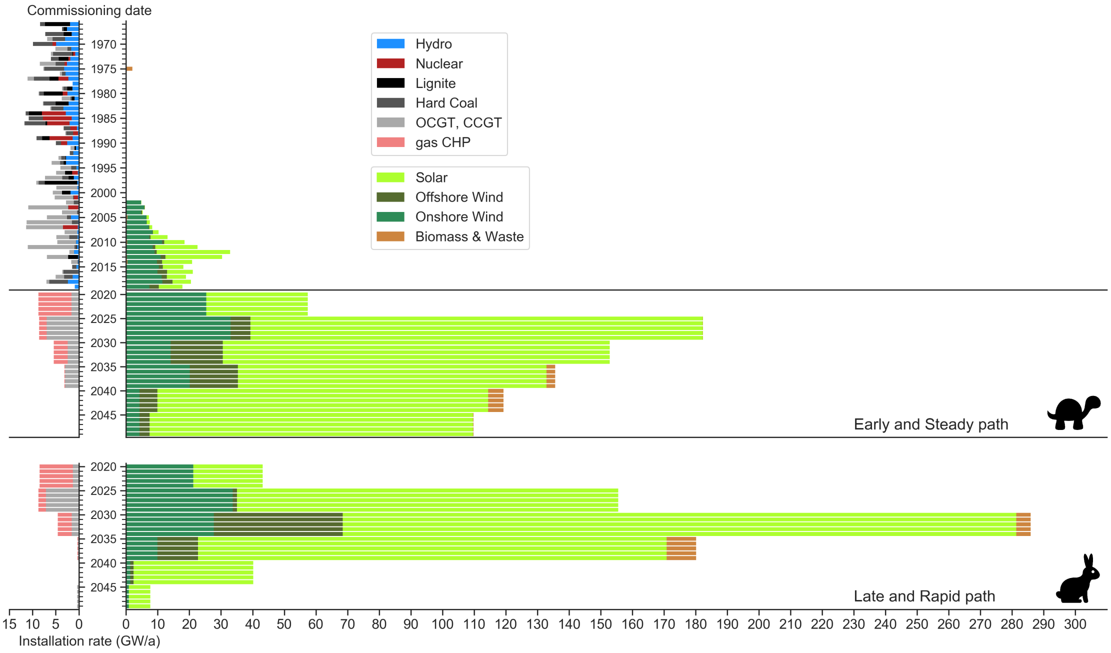

Supplementary Figure 22: Age distribution of European power plants in operation [3, 6] and required annual installation throughout the Early and Steady and Late and Rapid paths when the electricity, heating and transport sectors are included. .

Supplementary Notes.

1. CO_2 restriction paths with equivalent budget

The carbon budget from now onwards for the generation of electricity and the supply of heating in the residential and services sector in Europe accounts for 21 $GtCO_2$. It has been estimated based on a global carbon budget of 800 $GtCO_2$ to avoid temperature increments above 1.75°C relative to preindustrial period with a probability of more than 66% [7] [1]. The global budget is assumed to be split among regions according to a constant per-capita ratio which translates into a 6% share for Europe [8]. Out of the total emissions in Europe, the ratio corresponding to electricity and heating is considered constant and equal to present values. In 2017, electricity generation and heating in the residential and services sector emitted 1.56 $GtCO_2$ which represents 43.5% of European emissions, [1] and Supplementary Figure 1.

The B=21 $GtCO_2$ budget can be utilised following different transition paths. One option consists in assuming a linear CO_2 restriction path. Emissions will then reach zero in t_f

$$t_f = t_0 + \frac{2B}{e_0} \tag{1}$$

where t_0=2020, and e_0 represents the carbon emissions from electricity and heating sectors in 2020, which are assumed to be the same as in 2017.

Alternatively, emissions can be assumed to follow a path defined by one minus the cumulative distribution function (CDF_β) of a symmetric beta distribution in which $\beta_1 = \beta_2$.

$$\begin{aligned} e(t) &= e_0(1 - CDF_\beta(t)) \\ CDF_\beta(t) &= \int_{t_0}^{t} PDF_\beta(t)dt \\ PDF_\beta(t) &= \frac{\Gamma(2\beta_1)}{2\Gamma(\beta_1)}(t-t_0)^{\beta_1-1}(t_f-t)^{\beta_1-1} \end{aligned} \tag{2}$$

where Γ is the gamma function. The cumulative emissions fulfil $\int_{t_0}^{\infty} e(t)dt = B$.

The third option considered for the transition path is an exponential decay, following Raupach *et al.* [8]. In that case, emissions evolve as:

$$e(t) = e_0(1 + (r+m)t)e^{-mt} \tag{3}$$

where r is the initial linear growth rate, which here is assumed to be r=0, and the decay parameter m is determined by imposing the integral of the path to be equal to the budget.

$$\begin{aligned} B &= \int_{t_0}^{\infty} e_0(1 + (r+m)t)e^{-mt}dt \\ m &= \frac{1 + \sqrt{1 + \frac{rB}{e_0}}}{\frac{B}{e_0}} \end{aligned} \tag{4}$$

Although the exponential decay path approaches asymptotically to zero, we assume here that $e(2050) = 0$. By doing that, the final point of the different transition paths is equivalent and all of them achieve net-zero emissions in the electricity and heating sectors by 2050.

[1] Significant uncertainties in the estimated remaining carbon budget arise due to (a) uncertainties in the climate response to CO_2 and non-CO_2 emissions, (b) uncertainties in the level of historic warming, (c) potential additional carbon release from future permafrost thawing and methane release from wetlands, (d) level of non-CO_2 mitigation in the future [7].

2. Model description

In every time step, the optimisation objective, that is, the total annualised system cost is calculated as:

$$\min_{\substack{G_{n,s},E_{n,s},\\ F_\ell,g_{n,s,t}}} \left[\sum_{n,s} c_{n,s}\cdot G_{n,s} + \sum_{n,s}\hat{c}_{n,s}\cdot E_{n,s} + \sum_{\ell} c_\ell \cdot F_\ell + \sum_{n,s,t} o_{n,s,t}\cdot g_{n,s,t}\right]$$

where $c_{n,s}$ are the fixed annualised costs for generator and storage power capacity $G_{n,s}$ of technology s in every bus n, $\hat{c}_{n,s}$ are the fixed annualised costs for storage energy capacity $E_{n,s}$, c_ℓ are the fixed annualised costs for bus connectors F_ℓ, and $o_{n,s,t}$ are the variable costs for generation and storage dispatch $g_{n,s,t}$ in every hour t. Bus connectors ℓ include transmission lines but also converters between the buses implemented in every country (see Supplementary Figure 23), for instance, heat pumps that connect the electricity and heating bus.

The optimisation of the system is subject to several constraints. First, hourly demand $d_{n,t}$ in every bus n must be supplied by generators in that bus or imported from other buses. $f_{\ell,t}$ represents the energy flow on the link l and $\alpha_{n,\ell,t}$ indicates both the direction and the efficiency of flow on the bus connectors. $\alpha_{n,\ell,t}$ can be time-dependent such as in the case of heat pumps whose conversion efficiency depends on the ambient temperature.

$$\sum_s g_{n,s,t} + \sum_\ell \alpha_{n,\ell,t}\cdot f_{\ell,t} = d_{n,t} \quad \leftrightarrow \quad \lambda_{n,t} \quad \forall\, n,t \tag{5}$$

The Lagrange multiplier $\lambda_{n,t}$, also known as Karush-Kuhn-Tucker (KKT), associated with the demand constraint indicates the marginal price of the energy carrier in the bus n, *e.g.*, local marginal electricity price in the electricity bus.

Second, the maximum power flowing through the links is limited by their maximum physical capacity F_ℓ. For transmission links, $\underline{f}_{\ell,t} = -1$ and $\bar{f}_{\ell,t} = 1$, which allows both import and export between neighbouring countries. For a unidirectional converter *e.g.*, a heat resistor, $\underline{f}_{\ell,t} = 0$ and $\bar{f}_{\ell,t} = 1$ since a heat resistor can only convert electricity into heat.

$$\underline{f}_{\ell,t}\cdot F_\ell \le f_{\ell,t} \le \bar{f}_{\ell,t}\cdot F_\ell \qquad \forall\, \ell, t\ . \tag{6}$$

Third, for every hour the maximum capacity that can provide a generator or storage is bounded by the product between installed capacity $G_{n,s}$ and availabilities $\underline{g}_{n,s,t}$, $\bar{g}_{n,s,t}$. For instance, for solar generators $\underline{g}_{n,s,t}$ is zero and $\bar{g}_{n,s,t}$ refers to the capacity factor at time t

$$\underline{g}_{n,s,t}\cdot G_{n,s} \le g_{n,s,t} \le \bar{g}_{n,s,t}\cdot G_{n,s} \qquad \forall\, n,s,t\ . \tag{7}$$

The maximum power capacity for generators is limited by potentials $\bar{G}_{n,s}$ that are estimated taking into account physical and environmental constraints:

$$0 \le G_{n,s} \le \bar{G}_{n,s} \qquad \forall\, n,s\ . \tag{8}$$

The storage technologies have a charging efficiency η_{in} and rate $g^+_{n,s,t}$, a discharging efficiency η_{out} and rate $g^-_{n,s,t}$, possible inflow $g_{n,s,t,\text{inflow}}$ and spillage $g_{n,s,t,\text{spillage}}$, and standing loss η_0. The state of charge $e_{n,s,t}$ of every storage has to be consistent with charging and discharging in every hour and is limited by the energy capacity of the storage $E_{n,s}$. It should be remarked that the storage energy capacity $E_{n,s}$ can be optimised independently of the storage power capacity $G_{n,s}$.

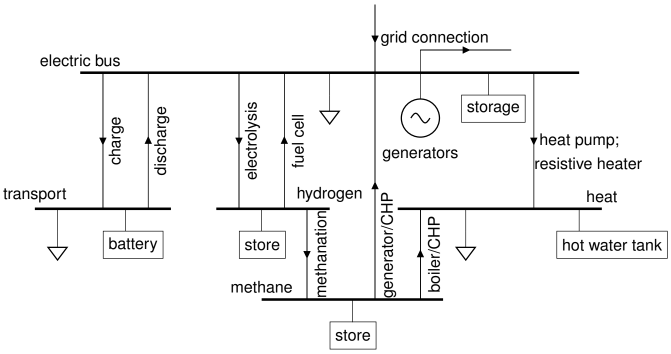

Supplementary Figure 23: Energy flow at a single node representing a country. Within each node, there is a bus (thick horizontal line) for every sector (electricity, transport and heating), to which different loads (triangles), energy sources (circles), storage units (rectangles) and converters (lines connecting buses) are attached. This is equivalent to the diagram shown in Fig. 7 in the main text.

$$e_{n,s,t} = \eta_0 \cdot e_{n,s,t-1} + \eta_{in}|g^+_{n,s,t}| - \eta^{-1}_{out}|g^-_{n,s,t}| + g_{n,s,t,\text{inflow}} - g_{n,s,t,\text{spillage}} \; ,$$
$$0 \leq e_{n,s,t} \leq E_{n,s} \qquad \forall\, n, s, t \; . \tag{9}$$

So far, Equations (5) to (9) represent mainly technical constraints but additional constraints can be imposed to bound the solution.

The interconnecting transmission expansion can be limited by a global constraint

$$\sum_\ell l_\ell \cdot F_\ell \leq \text{CAP}_{LV} \qquad \leftrightarrow \qquad \mu_{LV} \; , \tag{10}$$

where the sum of transmission capacities F_ℓ multiplied by the lengths l_ℓ is bounded by a transmission volume cap CAP_{LV}. In this case, the Lagrange/KKT multiplier μ_{LV} represents the shadow price of a marginal increase in transmission volume.

The maximum CO_2 allowed to be emitted by the system CAP_{CO2} can be imposed through the constraint

$$\sum_{n,s,t} \varepsilon_s \frac{g_{n,s,t}}{\eta_{n,s}} + \sum_{n,s} \varepsilon_s (e_{n,s,t=0} - e_{n,s,t=T}) \leq \text{CAP}_{CO2} \qquad \leftrightarrow \qquad \mu_{CO2} \tag{11}$$

where ε_s represents the specific emissions in CO_2-tonne-per-MWh$_\text{th}$ of the fuel s, $\eta_{n,s}$ the efficiency and $g_{n,s,t}$ the generators dispatch. In this case, the Lagrange/KKT multiplier represents the shadow price of CO_2, *i.e.*, the additional price that should be added for every unit of CO_2 to achieve the CO_2 reduction target in an open market.

2.1. Icon design acknowledgement

Icons used in Figures 1, 2, 4 and 8 in the main text were retrieved from the Noun Project [9] and are under a Creative Common license. We acknowledge the followings icons and authors: solar energy by fauzan akbar, wind energy by fauzan akbar, Air Conditioner by Arthur Shlain, Radiator by Nicolas LEULIET, Gas boiler by ProSymbols, cogeneration by Fabio Rinaldi, Water Tank by Luis Solorio, electric vehicle by Adrien Coquet, Dam by iconsmind.com, water heater by Stepan Voevodin, nitrogen by Edwin PM, Battery by notplayink!, transmission line by DARAYANI, Tank by Fabio Rinaldi, Methane by Michael Wohlwend, Hydropower by Georgiana Ionescu, Pellets by Viral faisalovers, Rabbit by Marco Galtarossa, tortoise by Christopher T. Howlett.

The map shown in Figure 6 in the main text was created using Cartopy [10].

3. Sectors description and data

3.1. Electricity sector

Hourly electricity demand for every country corresponding to 2015 is retrieved from EU Network Transmission System Operators of Electricity (ENTSO-E) via the convenient dataset prepared by the Open Power System Data (OPSD) initiative [11]. In every country, electricity can be generated by solar PV, onshore wind, offshore wind, Open Cycle Gas Turbines (OCGT), Combined Cycle Gas Turbines (CCGT), coal, lignite, and nuclear power plants and CHP units using either gas, coal or biomass. Their costs, lifetimes and efficiencies are shown in Supplementary Tables 4 and 5. To represent scheduled shut-downs, a constant 90% availability is assumed for nuclear power plants.

Country-wise onshore and offshore wind capacity factor time series are modelled by converting wind velocity from Climate Forecast System Reanalysis (CFSR) [12] into wind generation, following the methodology described in [13]. CFSR database comprises hourly resolution and spatial resolution equal to 0.3125°x0.3125°, which in Europe roughly corresponds to 40x40 km^2. For every country, a capacity layout proportional to wind resource is assumed. Following [14], large countries are divided into up to 4 regions sorted by wind resource. Independent classes of generators with different time series and average full load hours are added to a single node representing a country. Their optimised capacities are later aggregated on a country level for analysis.

Time series representing the hourly capacity factors for solar PV were obtained by converting bias-corrected CFSR reanalysis irradiance into solar electricity generation, assuming a uniform capacity layout across every country. Details on the conversion and aggregation methodology can be found in [15], the complete time series dataset is available in 10.5281/zenodo.1321809. 50% of PV capacity in every country is assumed to be utility-scale installations and 50% rooftop PV systems with the cost and characteristics gathered in Supplementary Tables 4 and 5. The discount rate is assumed to be 7% for the former and 4% for the latter. As discussed in [16] the impact of this assumption is limited.

The maximum capacity for onshore wind, offshore wind, and solar PV that can be installed in every country is limited by the estimated potentials. Those are determined by summing the available land in every reanalysis grid cell, which in turn is calculated by considering only the suitable land for every technology, according to the Corine Land Cover (CLC) database [17] and subtracting Natura 2000 protected areas [18]. For onshore and offshore wind, the potential is calculated as 20% of the available land. Conversion factors of 10 MW/km^2 and 145 MW/km^2 are considered for wind and solar respectively. For solar PV, 3% of the available land is used for estimating the potential.

$$Potential_{n,PV} = \sum_i 0.03(A_i^{CLC,PV} - A_i^{Natura2000}) \quad \text{for} \quad i \in n \tag{12}$$

where $A_i^{CLC,PV}$ is the area of the grid cell belonging to PV categories in the CLC database, Supplementary Table 1, and $A_i^{Natura2000}$ is the area of the grid cell protected by the Natura 2000 network.

$$Potential_{n,wind} = \sum_i 0.2(A_i^{CLC,wind} - A_i^{Natura2000})k_n \quad \text{for} \quad i \in n \tag{13}$$

For wind, k_n is a coefficient calculated by imposing the condition that in none of the grid cells the installed capacity surpasses the potential. This represents a conservative approach. Higher potentials could be attained if assumed capacity layout is not proportional to the wind resource. For offshore wind, only areas whose sea depth is lower than 50 m are considered as valid.

Reservoir hydropower and run-of-river capacities are exogenously fixed at their values in 2015. Hourly inflow is modelled based on rainfall in the CFSR data set as described in [20]. CHP units are modelled as extraction condensing units, the feasible space representing the possible combinations of power and heat outputs is included as a constraint in the model, as detailed in [20]. Electricity can be stored in static batteries, hydrogen storage and Pumped Hydro Storage (PHS). The capacity of the later in every country is exogenously fixed at 2015 values. Alkaline electrolysers are assumed since they have a lower cost [21] and

Supplementary Table 1: Land types considered suitable for every technology. Categories in Corine Land Cover database [17] are selected following [19].

Solar PV	artificial surfaces (1-11), agriculture land except for those areas already occupied by agriculture with significant natural vegetation and agro-forestry areas (12-20), natural grasslands (26), bare rocks (31), and sparsely vegetated areas (32)
Onshore wind	agriculture areas (12-22), forests (23-25), scrubs and herbaceous vegetation associations (26-29), bare rocks (31), and sparsely vegetated areas (32)
Offshore wind	sea and ocean (44)

higher cumulative installed capacity [22] than PEM electrolysers. Hydrogen can be stored in overground steel tanks or underground salt caverns [22]. For the latter, energy capacities in every country are limited to the potential estimation for onshore salt caverns within 50 km of shore to avoid environmental issues associated with brine solution disposal, see Figure 7 in [23]. Electricity can also be used to produce methane by combining hydrogen and direct air captured (DAC) CO_2 in the Sabatier reaction. Following [20], the energy consumed in DAC is taken into account by reducing the efficiency of the Sabatier reaction to 60%. Alternative CO_2 sources, such as capturing industry process emissions or biomass-related emissions, are not included.

The transmission links between countries are assumed to be high-voltage direct current (HVDC) connections. The lengths l_ℓ are set by the distance between the geographical mid-points of each country so that some of the transmission within each country is also reflected in the optimisation. A factor of 25% is added to the line lengths to account for the fact that transmission lines cannot be placed as the crow flies due to land use restriction. For the transmission line capacities F_ℓ, a safety margin of 33% of the installed capacity is used to satisfy n-1 requirements [24]. For 2020 and 2030, the capacities correspond to the values assumed in the ENTSOE Ten-Year Network Development Plan (TNYDP), see Supplementary Table 2 and [25]. The values for 2025 are interpolated assuming a linear capacity expansion between 2020 and 2030 for every link. For years from 2035 onwards, capacities are fixed at 2030 values.

Supplementary Table 2: Transmission capacities (MW) for interconnections [25].

Link	2020	2030	Link	2020	2030	Link	2020	2030
AL-GR	250	250	FI-EE	1000	1000	LU-FR	0	0
AL-ME	350	350	FI-NO	0	0	LV-EE	1600	1600
AL-MK	200	200	FI-SE	2300	2800	LV-LT	1200	1800
AL-RS	760	760	FR-BE	4300	4300	ME-AL	350	350
AT-CH	1700	1700	FR-CH	3700	3700	ME-BA	400	400
AT-CZ	1000	1000	FR-DE	3000	4800	ME-IT	1200	1200
AT-DE	5000	7500	FR-ES	5000	8000	ME-RS	1000	1000
AT-HU	1200	1200	FR-GB	5400	5400	MK-AL	200	200
AT-IT	555	1655	FR-IE	0	700	MK-BG	150	150
AT-SI	1200	1200	FR-IT	4350	4350	MK-GR	400	400
BA-HR	1344	1844	FR-LU	380	380	MK-RS	1050	1050
BA-ME	500	500	GB-BE	1000	1000	NI-GB	80	500
BA-RS	1100	1100	GB-DK	1400	1400	NI-IE	1100	1100
BE-DE	1000	1000	GB-FR	5400	5400	NL-BE	2400	2400
BE-FR	2800	2800	GB-IE	500	500	NL-DE	4450	5000
BE-GB	1000	1000	GB-IS	0	0	NL-DK	700	700
BE-LU	1080	1080	GB-NI	500	500	NL-GB	1000	1000
BE-NL	2400	2400	GB-NL	1000	1000	NL-NO	700	700
BG-GR	1728	1728	GB-NO	1400	1400	NO-DE	1400	1400
BG-MK	530	530	GR-AL	250	250	NO-DK	1640	1640
BG-RO	1400	1400	GR-BG	1032	1032	NO-FI	0	0
BG-RS	600	600	GR-CY	2000	2000	NO-GB	1400	1400
CH-AT	1700	1700	GR-IT	500	500	NO-NL	700	700
CH-DE	4700	4700	GR-MK	350	350	NO-SE	3695	3695
CH-FR	1300	1300	HR-BA	1312	1812	PL-CZ	600	600
CH-IT	6240	6240	HR-HU	2000	2000	PL-DE	3000	3000
CY-GR	2000	2000	HR-IT	0	0	PL-DK	0	0
CZ-AT	1200	1200	HR-RS	600	600	PL-LT	1000	1000
CZ-DE	2100	2600	HR-SI	2000	2000	PL-PL	5000	5000
CZ-PL	500	500	HU-AT	800	800	PL-SE	600	600
CZ-SK	2100	2100	HU-HR	2000	2000	PL-SK	990	990
DE-AT	5000	7500	HU-RO	1300	1300	PT-ES	3500	3500
DE-BE	1000	1000	HU-RS	600	600	RO-BG	1500	1500
DE-CH	3286	3286	HU-SI	1700	1700	RO-HU	1400	1400
DE-CZ	1500	2000	HU-SK	2000	2000	RO-RS	1450	1450
DE-DK	4000	4000	IE-FR	0	700	RS-AL	330	330
DE-FR	3000	4800	IE-GB	500	500	RS-BA	1200	1200
DE-LU	2300	2300	IE-NI	1100	1100	RS-BG	350	350
DE-NL	4450	5000	IS-GB	0	0	RS-HR	600	600
DE-NO	1400	1400	IT-AT	385	1385	RS-HU	600	600
DE-PL	2000	2000	IT-CH	3860	3860	RS-ME	1100	1100
DE-SE	615	1315	IT-FR	2160	2160	RS-MK	950	950
DK-DE	4000	4000	IT-GR	500	500	RS-RO	1050	1050
DK-DK	1200	1200	IT-HR	0	0	SE-DE	615	1315
DK-GB	1400	1400	IT-IT	5750	5750	SE-DK	1980	1980
DK-NL	700	700	IT-ME	1200	1200	SE-FI	2400	3200
DK-NO	1640	1640	IT-SI	1380	1380	SE-LT	700	700
DK-PL	0	0	IT-TN	0	0	SE-NO	3995	3995
DK-SE	2440	2440	LT-LV	1500	2100	SE-PL	600	600
EE-FI	1016	1016	LT-PL	1000	1000	SI-AT	1200	1200
EE-LV	1600	1600	LT-SE	700	700	SI-HR	2000	2000
ES-FR	5000	8000	LU-BE	700	700	SI-HU	2000	2000
ES-PT	4200	4200	LU-DE	2300	2300	SI-IT	1530	1530

3.2. Heating sector

Annual heat demands for European countries are retrieved from [26]. They are converted into hourly heat demand based on the population-weighted [27] Heating Degree Hour (HDH), that is, heating is assumed to be proportional to the difference between ambient temperature and a threshold temperature. 17°C is assumed as threshold temperature. Ambient temperature is read from the same reanalysis

database [12] used to model wind and solar PV time series. The estimated values for total annual demand in Europe are similar for electricity and heating, accounting for 2854 TWh$_{el}$/a and 3562 TWh$_{th}$/a respectively but heating demand shows a much more pronounced seasonal variation, see Supplementary Figure 24.

For every country, heating demand is split between low-population density areas and high-population density areas. 44.6% of the European population is estimated to live in the latter [20] where district heating systems can be deployed. In high-density population areas, heating can be supplied by central ground-sourced heat pumps, heat resistors and gas boilers, as well as by CHP units. All the previous technologies are assumed to be integrated into district heating networks. Furthermore, individual air-sourced heat pumps are also allowed in those areas. In low-density population areas, heating can be supplied by individual ground-sourced heat pumps, heat resistors and gas boilers. Costs, lifetimes, and efficiencies of the different technologies are included in Supplementary Tables 4 and 5.

The Coefficient of Performance (COP) of heat-pumps depends on ambient or ground temperature to capture the lower COP in winter. COP depends on the difference between the source and the sink temperatures $\Delta T = T_{sink} - T_{source}$. For air-sourced heat pumps (ASHP), $COP = 6.81 + 0.121\Delta T + 0.000630\Delta T^2$, for ground-sourced heat pumps (GSHP), $COP = 8.77 + 0.150\Delta T + 0.000734\Delta T^2$ [28]. The sink water temperature is assumed to be $T_{sink} = 55°$C, the source temperature for air and ground is taken from the same reanalysis database used to estimate heating demand [12]. Thermal energy can be stored in large water pits associated with district heating systems and individual thermal energy storage (TES), *i.e.*, small water tanks. A thermal energy density of 46.8 kWh$_{th}$/m^3 is assumed, corresponding to a temperature difference of 40 K. The decay of thermal energy $1 - \exp(-\frac{1}{24\tau})$ is assumed to have a time constant of τ=180 days for central TES and τ=3 days for individual TES. Charging and discharging efficiencies are 90% due to pipe losses.

Capacities already existing for technologies supplying heat are retrieved from [29]. For the sake of simplicity, coal, oil and gas boilers capacities are assimilated to gas boilers. Besides that, existing capacities for heat resistors, ASHP, and GSHP are included in the model. For high-density population areas, the penetration of district-heating is assumed to remain fixed at 2015 values, [30] and Supplementary Table 3. Cooling demand is currently supplied by electricity so it is included in the electricity demand time series. It is assumed to remain constant throughout the paths. For a thorough discussion of the impact of changing cooling demand, the reader is referred to [31].

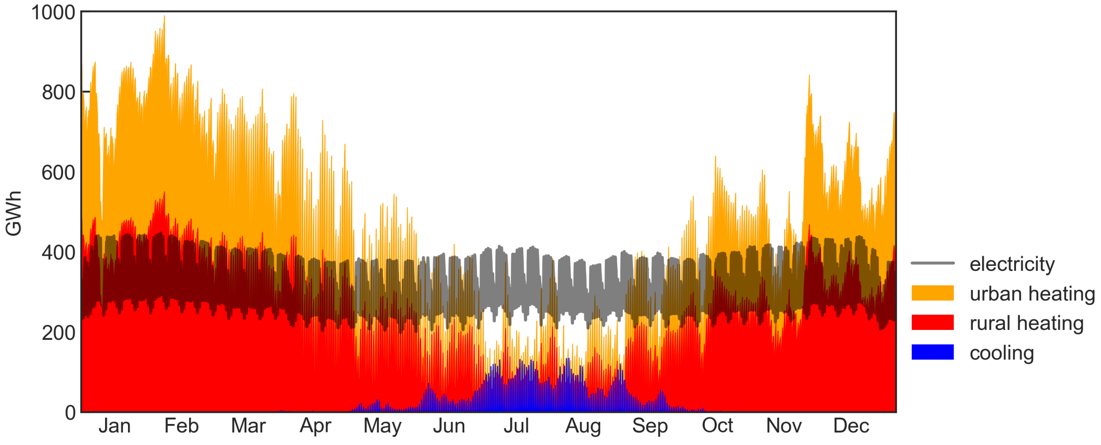

Supplementary Figure 24: Electricity, rural and urban heating, and cooling demands for Europe.

3.3. Biomass

Solid biomass can be burnt in CHP or central heating plants associated with district heating systems or in power plants to produce electricity. The model does not include biogas that could be burnt or upgraded

Supplementary Table 3: Current penetration of district heating in European countries [30].

Country	District heating penetration
AT	0.14
BA	0.0
BE	0.0
BG	0.16
CH	0.04
CZ	0.4
DE	0.14
DK	0.64
EE	0.52
ES	0.0
FI	0.39
FR	0.06
GB	0.02
GR	0.0
HR	0.07
HU	0.12
IE	0.0
IT	0.03
LT	0.56
LU	0.0
LV	0.3
NL	0.04
NO	0.03
PL	0.41
PT	0.0
RO	0.23
RS	0.27
SE	0.51
SI	0.09
SK	0.54

into biomethane. A conservative approach is followed to estimate biomass potentials in every country. From the JRC-ENSPRESO database [32, 33], the potential estimations for 2030 in the scenario ‘medium’ are retrieved, but only the types of biomass which are not competing with crops are considered valid. In essence, biomass potentials include only the following items: primary agricultural residues, primary and secondary forestry energy residues including sawdust, forestry residues from landscape care, and municipal waste.

3.4. Existing power plants and decommissioning

For conventional technologies, *i.e.* OCGT, CCGT, coal, lignite, nuclear and gas CHP, installed capacities in every country in 2020 and commissioning dates are retrieved from [6]. A two-step method was implemented to fill commissioning date for power plants whose data was missing. First, for units larger than 50 MW, commissioning dates have been searched and manually added. Then, for smaller units, a Kernel Density Estimation (KDE) approach is used. In essence, for every technology and country, the units with available data are used to create a distribution, which is then used to assign an estimated commissioning date for those units with missing data. For solar PV, the installed capacities in 2020 and the installation dates were obtained by processing annual installed capacities statistics from [3]. For offshore and onshore wind, capacities and age are retrieved from [34]. Existing power plants are assumed to be decommissioned at their corresponding commissioning date plus lifetime (Supplementary Table 5). When a power plant has been retrofitted, we assume that its operating life is extended by half of its nominal lifetime. For heating capacities, 25% of existing capacities in 2015 are assumed to be decommissioned in every 5-year time step after 2020.

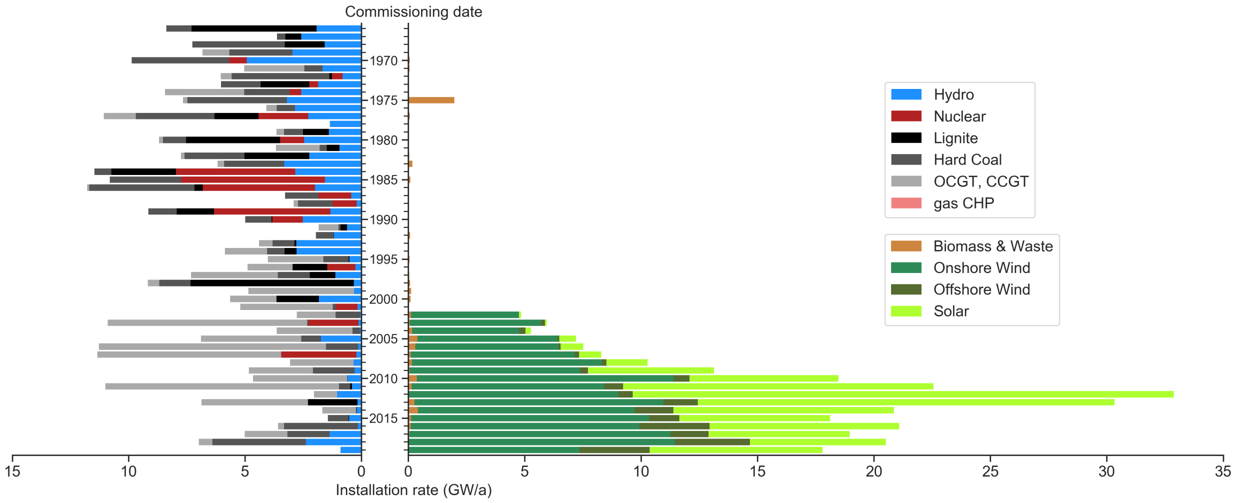

Supplementary Figure 25: Age distribution of European power plants in operation [3, 6].

3.5. *Transport sector*

The transport sector is included only in the final analysis of the paper. In that case, road and rail transport are considered to be electrified at a rate equal to the CO_2 reduction in the heating and electricity sectors relative to 2020. In this way, transport-related CO_2 emissions sink in parallel to the other sectors. Annual energy demands from road and rail transport for every country are retrieved from [35]. Aviation, shipping, and pipe transport are not included in the model. A country-specific factor (averaging 3.5) is used to account for the increased efficiency when electrifying transport. Country-specific factors are computed by comparing the current car final energy consumption per km in [35] (averaging 0.7 kWh/km) to the 0.2 kWh/km value assumed for plug-to-wheels efficiency in EVs. The characteristic weakly profile provided by the German Federal Highway Research Institute (BASt) [36] is used to obtain hourly time series for European countries taking into account the corresponding local times. Furthermore, a temperature dependence is included in the time series to account for heating/cooling demand in transport. For temperatures below/above 15°C/20°C, temperature coefficients of 0.63%/°C and 0.98%/°C are assumed, see [20] for more details. When fully electrified, the annual electricity demand from transport sector in Europe accounts for 1,102 TWh/a.

At every time step, the internal-combustion vehicles transformed into battery electric vehicles (BEV) are assumed to include a battery with a storage capacity of 50 kWh, charging capacity of 11 kW, and 90% charging efficiency. It is considered that half of the existing BEV of them can shift their charging time as well as discharge into the grid to facilitate the operation of the system and reduce its total cost. Furthermore, it is assumed that, at every time step, 25% of the existing BEV can provide vehicle-to-grid (v2g) services. The BEV state of charge is forced to be higher than 75% at 5 a.m. every day, through $e_{n,s,t}$ in Equation (9), to ensure that the batteries are full in the morning peak usage. This also restricts BEV demand to be shifted within a day and prevent EV batteries from becoming seasonal storage. The percentage of BEV connected to the grid at any time is inversely proportional to the transport demand profile, which translates into an average/minimum availability of 80%/62%. This approach is conservative compared to most of the literature. For instance, in [37] the average parking time of the European fleet of vehicles is estimated at 92%. The cost of the EV batteries is not included in the model since it is assumed that EV owners buy them to satisfy their mobility needs.

3.6. *Levelised Cost of Energy (LCOE)*

The Levelised Cost of Energy is defined as the total system cost per unit of consumed energy, that is, including supplied electricity and heating demand.

4. Cost assumptions

Supplementary Table 4: Overnight investment cost assumptions per technology and year. All costs are given in real 2015 money.

Technology	Unit	2020	2025	2030	2035	2040	2045	2050	source
Onshore Wind	€/kW$_{el}$	1118	1077	1035	1006	977	970	963	[21]
Offshore Wind	€/kW$_{el}$	2128	2031	1934	1871	1808	1792	1777	[21]
Solar PV (utility-scale)[a]	€/kW$_{el}$	398	326	254	221	188	169	151	[38]
Solar PV (rooftop)[a]	€/kW$_{el}$	1127	955	784	723	661	600	539	[39]
OCGT	€/kW$_{el}$	453	444	435	429	423	417	411	[21]
CCGT	€/kW$_{el}$	880	855	830	822	815	807	800	[21]
Coal power plant	€/kW$_{el}$	3845	3845	3845	3845	3845	3845	3845	[40]
Lignite	€/kW$_{el}$	3845	3845	3845	3845	3845	3845	3845	[40]
Nuclear	€/kW$_{el}$	7940	7940	7940	7940	7940	7940	7940	[40]
Reservoir hydro	€/kW$_{el}$	2208	2208	2208	2208	2208	2208	2208	[41]
Run of river	€/kW$_{el}$	3312	3312	3312	3312	3312	3312	3312	[41]
PHS	€/kW$_{el}$	2208	2208	2208	2208	2208	2208	2208	[41]
Gas CHP	€/kW$_{el}$	590	575	560	550	540	530	520	[21]
Biomass CHP	€/kW$_{el}$	3500	3400	3300	3224	3150	3075	3000	[21]
Coal CHP	€/kW$_{el}$	1900	1880	1860	1841	1822	1803	1783	[21]
Biomass central heat plant	€/kW$_{el}$	890	865	840	820	800	780	760	[21]
Biomass power plant	€/kW$_{el}$	3500	3400	3300	3224	3150	3075	3000	[21]
HVDC overhead	€/MWkm	400	400	400	400	400	400	400	[42]
HVDC inverter pair	€/MW	150000	150000	150000	150000	150000	150000	150000	[42]
Battery storage	€/kWh	232	187	142	118	94	84	75	[21]
Battery inverter	€/kW$_{el}$	270	215	160	130	100	80	60	[21]
Electrolysis	€/kW$_{el}$	600	575	550	537	525	512	500	[21]
Fuel cell	€/kW$_{el}$	1300	1200	1100	1025	950	875	800	[21]
H_2 storage underground	€/kWh	3.0	2.5	2.0	1.8	1.5	1.4	1.2	[21]
H_2 storage tank	€/kWh	57	50	44	35	27	24	21	[21]
DAC (direct-air capture)	€/(tCO_2/a)	772	577	383	317	251	230	210	[43]
Methanation	€/kW$_{H_2}$	1000	1000	1000	1000	1000	1000	1000	[44]
Central gas boiler	€/kW$_{th}$	60	55	50	50	50	50	50	[21]
Decentral gas boiler	€/kW$_{th}$	312	304	296	289	282	275	268	[21]
Central resistive heater	€/kW$_{th}$	70	65	60	60	60	60	60	[21]
Decentral resistive heater	€/kW$_{th}$	100	100	100	100	100	100	100	[44]
Central water tank storage	€/kWh	0.6	0.6	0.5	0.5	0.5	0.5	0.5	[21]
Decentral water tank storage	€/kWh	18	18	18	18	18	18	18	[21, 45]
Decentral air-sourced heat pump	€/kW$_{th}$	940	894	850	827	804	782	760	[21]
Central ground-sourced heat pump	€/kW$_{th}$	657	625	592	577	562	547	532	[21]
Decentral ground-sourced heat pump	€/kW$_{th}$	1500	1450	1400	1349	1299	1250	1200	[21]

[a] In the reference, solar PV investment cost is expressed in 2019-euros. It has been translated into 2015-euros, assuming a growth rate of 2%.

Supplementary Table 5: Efficiency, lifetime and FOM cost per technology (values shown corresponds to 2020).

Technology	FOM[a] [%/a]	Lifetime [a]	Efficiency	Source
Onshore Wind	1.3	27		[21]
Offshore Wind	1.9	27		[21]
Solar PV (utility-scale)	3.0	30		[38]
Solar PV (rooftop)	2.0	30		[39]
OCGT	1.8	25	0.42	[21]
CCGT	3.3	25	0.59	[21]
Coal power plant	1.6	40	0.33	[40]
Lignite	1.6	40	0.33	[40]
Nuclear	1.4	40	0.33	[40]
Reservoir hydro[b]	1.0	80	0.9	[41]
Run of river[b]	2.0	80	0.9	[41]
PHS[b]	1.0	80	0.75	[41]
Gas CHP[c]	3.3	25		[21]
Biomass CHP[c]	3.6	25		[21]
Coal CHP[c]	1.6	25	1.0	[21]
Biomass central heat plant	5.8	25	1.0	[21]
Biomass power plant	3.6	25	0.31	[21]
HVDC overhead	2.0	40		[42]
HVDC inverter pair	2.0	40		[42]
Battery storage	0.0	20		[21]
Battery inverter	0.2	20	0.9	[21]
Electrolysis	5.0	25	0.8	[21, 46]
Fuel cell	5.0	10	0.58	[21, 46]
H_2 storage underground	2.0	100	1.0	[21]
H_2 storage tank	1.1	25		[21]
DAC (direct-air capture)[e]	4.0	30		[43]
Methanation	3.0	25	0.6	[44]
Central gas boiler	3.2	25	1.0	[21]
Decentral gas boiler	6.6	20	0.97	[21]
Central resistive heater	1.5	20	0.99	[21]
Decentral resistive heater	2.0	20	0.9	[44]
Central water tank storage	0.5	20		[21]
Decentral water tank storage	1.0	20		[21, 45]
Water tank charger/discharger			0.9	
Decentral air-sourced heat pump[d]	3.0	18		[21]
Central ground-sourced heat pump[d]	0.3	25		[21]
Decentral ground-sourced heat pump[d]	1.9	20		[21]

[a] Fixed Operation and Maintenance (FOM) costs are given as a percentage of the overnight cost per year.
[b] Hydroelectric facilities are not expanded in this model and are considered to be fully amortized.
[c] Efficiency for Combined Heat and Power (CHP) plants depends on the electricity/heat output and it is modelled as described in the text.
[d] Coefficient of performance (COP) of heat pumps is modelled as a function of temperature, as described in the text.
[e] Investments in methanation and DAC are not allowed independently, only together as 'Methanation+DAC', see text.

Supplementary Table 6: Costs and emissions coefficient of fuels.

Fuel	Cost [€/MWh$_{th}$]	Source	Emissions [tCO_2/MWh$_{th}$]	Source
coal	8.2	[47]	0.336	[48]
lignite	2.9	[41]	0.407	[48]
gas	20.1	[47]	0.201	[48]
oil	50.0	[49]	0.266	[48]
nuclear	2.6	[40]	0	
solid biomass[a]	25.2	[32, 50]	0	

[a] Raw biomass fuel cost is assumed as the middle value of the range provided in the references for different European countries and types of sustainable biomass.

[b] We neglect the contribution from emissions embedded in infrastructure additions as we assume that they are significantly smaller than those coming from fossil-fuels combustion. As the energy system decarbonise the CO_2 embedded in wind turbines and solar panels will be even further reduced.

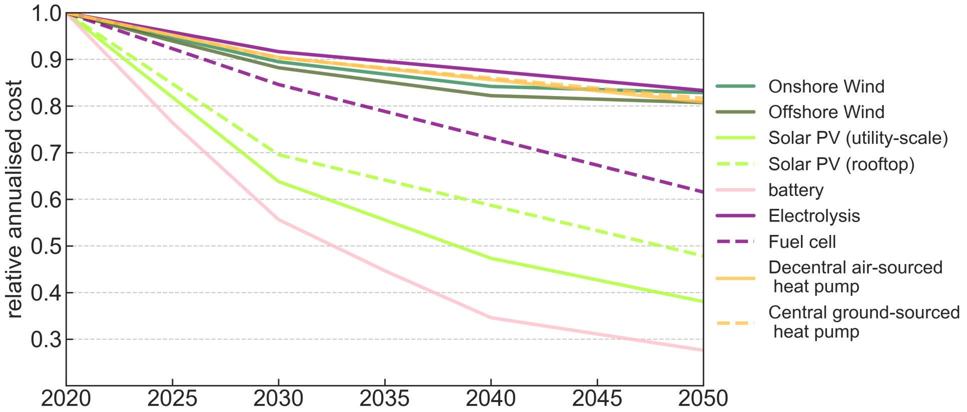

Supplementary Figure 26: Evolution of annualised costs, relative to 2020, for some selected technologies.

4.1. *Distribution networks*

Half of the solar PV capacity is assumed to be installed in rooftops. Consequently, the PV deployment that we observe will require extending and increasing the capacity of electricity distribution networks. The deployment of battery electric vehicles (BVEs) also requires enhancing the distribution networks. Distributed PV generation will contribute to charging BEVs and this could reduce the need for an increased distribution capacity. The cost of extending distribution networks is estimated in 140 €/kW$_{PV}$, with 30 years lifetime and 3% FOM [21, 51]. In the model, the cost of expanding and maintaining distribution networks are not included in the optimisation but calculated based on the optimal PV capacity and added ex-post. For the Early and Steady path, distribution networks costs represent 140 B€, that is, 2.6% of the cumulative system cost. District heating and gas distribution networks are not included in the optimisation, but the cost of expanding those networks is estimated and compared with the system costs in the scenarios with and without district heating expansion, see Section 4.5.

4.2. *Up-to-date cost assumptions for solar PV*

The combination of rapid learning with faster-than-expected capacity deployment has led to lower-than-expected costs for solar PV. The potential of this technology has been repeatedly underestimated by the International Energy Agency [52], Greenpeace [53], and PV scientists [54]. The investment cost for utility-scale solar PV in 2020 is estimated in the range of 398-423 €/kW [21, 39]. For rooftop PV installations in Europe, a wider range is found, 1070-1127 €/kW [21, 39, 55], due to the higher impact of local experience and labour costs. Solar PV is forecast to achieve an investment cost between 151 €/kW [38] and 241

€/kW [21] for large installations in 2050.

Assuming outdated costs for solar PV is also known to be a flaw of most Integrated Assessment Models (IAMs) and it can have a huge impact on the results. Creutzig *et al.* already pointed out this problem in [53] where they found similar solar PV penetration to ours when up-to-date costs are assumed for solar PV. Breyer and co-workers have also emphasized the key role that solar PV plays in decarbonisation paths in Europe [56] and globally [57] when proper costs are assumed. However, the problem persists. For instance, the PRIMES model used in the report supporting the *Clean Planet for All* strategy of the EU Commission [58] assumes 407-495 €/kW in 2050 [59], which is higher than the lower range value for today's costs. Even more worrying are the findings by Krey *et al.* [60]. The authors review the techno-economic assumptions in the electricity sector among fifteen different global and national IAMs. Figure 4 in [60] shows that most of the reviewed IAMs include cost assumptions for solar PV in 2050 close to 1000 €/kW. Although they do not specify if the cost refers to utility-scale or rooftop installations, the values are twice as high as the cost already achieved by this technology in large installations.

4.3. Discount rate

We use a financial discount rate to annualise the cost of every asset including generation, storage, and transmission. The financial discount rate is equal to 7% for all the investment, except decentral assets such as rooftop PV or individual water tanks for which 4% is assumed. Discount rates are influenced by the economic situation in a country and this could strongly impact the results [61]. Moreover, the accumulated experience with a certain technology, globally and inside the country, affects the perceived risks and the cost of capital for that technology. When investigating transition paths, other authors have considered sector-specific discount rates although they do not update them as the technology becomes more mature [58]. It is extremely difficult to meaningfully estimate country-specific discount rates for future years. Hence, we have chosen a constant 7% discount rate for all the technologies and countries. Schyska and Kies have assessed the impacts of different cost of capital on the optimal European power system [62].

Besides the financial discount rate, a different social discount rate is used to calculate the cumulative system cost. This is common practice when comparing transition paths derived from IAMs and energy models [58, 63]. We have selected a social discount rate of 2%, which is similar to the economic growth in the European Union, that averaged 1.6% in the past 20 years. This is in agreement with reference [64], in which the impact of discount rates for emissions pathways and negative emissions are analysed. The authors recommend using low social discount rates, around 2%.

4.4. Jobs creation

Currently, the total number of renewable energy jobs in member states of the European Union is estimated at 1.2 million [65]. In [66], a systematic review of literature is conducted and the average number of full-time-equivalent (FTE) jobs associated with every renewable technology is provided. FTE jobs include both installation as well as operation and maintenance jobs. Using that data, and assuming 40-year work life, we have estimated the newly created jobs associated with the expansion of solar PV, wind and biomass capacities. Cumulative new jobs represent 1.9 and 1.8 million for the Early and Steady and Late and Rapid path respectively. This is in agreement with the analysis included in the *Clean Energy for All* strategy [58] which estimates the creation of 2.1 million jobs under the 1.5°C scenario. As other technological sectors, renewables suffer from a major gender imbalance. The transition is expected to have a positive impact on this aspect since women represent 32% of the renewable energy workforce while they only account for 22% of the workforce in the oil and gas industry [67].

Supplementary Table 7: Technology assumptions and average jobs creation based on the systematic literature review in [66].

technology	lifetime (years)	annual CF	full-time-equivalent jobs (jobs/GWh)
solar PV (rooftop)	25	0.11	2
solar PV (utility-scale)	25	0.11	0.5
wind	20	0.33	0.5
biomass	30	0.8	0.2

4.5. *Transition paths including district heating expansion*

A wide range of cost estimation for DH system is found in the literature: 107 €/kW$_{th}$ for 2030 [59], 220 €/kW$_{th}$ [45], 400 €/kW$_{th}$ [51]. Assuming 40 years lifetime, 1% FOM [51], 4% discount rate, and considering that the peak in urban heat demand is approximately 500 GW$_{th}$, the required DH expansion and maintenance represents between 6.5 and 24 B€/a, which could be offset by the system cost reduction. Furthermore, the avoided expansion of gas distribution networks when DH is deployed will also contribute to offsetting DH costs.

4.6. *Transition paths assuming heating demand reduction due to building renovation*

Assuming a 2% reduction of space heating per year, constant hot water demand, and neglecting any rebound effect will translate into an approximate decrease of 40% of heating demand at the end of the path. Hence, heating demand in 2050 would represent 2,137 TWh$_{th}$/a which is similar to the Baseline scenario in the *Clean Planet for All* analysis, which assumes 2,208 TWh$_{th}$/a in 2050 [58]. Cumulative system cost decreases by 760 B€ compared to the paths with constant heating demand. If the cost for heat savings due to building renovation is assumed to be 100 €/MWh (average for the renovation costs in [20]), the total cost for building renovation represents 142 B€, which is significantly offset by the savings in cumulative system cost.

References

[1] National emissions reported to the UNFCCC and to the EU Greenhouse Gas Monitoring Mechanism , EEA. URL https://www.eea.europa.eu/data-and-maps/data/national-emissions-reported-to-the-unfccc-and-to-the-eu-greenhouse-gas-monitoring-mecha

[2] Mantzos, L. *et al.* JRC-IDEES: Integrated Database of the European Energy Sector (2017). URL http://www.sciencedirect.com/science/article/pii/S0360544216310295.

[3] Renewable Capacity Statistics 2019, IRENA. URL https://www.irena.org/publications/2019/Mar/Renewable-Capacity-Statistics-2019.

[4] Photovoltaics Report. Tech. Rep., Fraunhofer ISE (2019). URL https://www.ise.fraunhofer.de/content/dam/ise/de/documents/publications/studies/Photovoltaics-Report.pdf.

[5] Victoria, M., Gallego, C., Anton, I. & Sala, G. Past, Present and Future of Feed-in Tariffs in Spain: What are their Real Costs? *27th European Photovoltaic Solar Energy Conference and Exhibition* 4612–4616 (2012). URL http://www.eupvsec-proceedings.com/proceedings?paper=17736.

[6] Gotzens, F., Heinrichs, H., Hörsch, J. & Hofmann, F. Performing energy modelling exercises in a transparent way - The issue of data quality in power plant databases. *Energy Strategy Reviews* **23**, 1–12 (2019). URL http://www.sciencedirect.com/science/article/pii/S2211467X18301056.

[7] Global Warming of 1.5°C, Intergovernmental Panel on Climate Change (IPCC). Tech. Rep. (2018). URL https://www.ipcc.ch/sr15/.

[8] Raupach, M. R. *et al.* Sharing a quota on cumulative carbon emissions. *Nature Climate Change* **4**, 873–879 (2014). URL https://www.nature.com/articles/nclimate2384.

[9] The noun project. URL https://thenounproject.com/.

[10] Met Office. *Cartopy: a cartographic python library with a Matplotlib interface*. Exeter, Devon (2010 - 2015). URL https://scitools.org.uk/cartopy.

[11] Open Power System Data. 2018. Data Package Time series. Version 2018-03-13. (Primary data from various sources, for a complete list see URL). URL https://data.open-power-system-data.org/time_series/2018-03-13/.

[12] Saha, S. *et al.* NCEP climate forecast system version 2 (CFSv2) selected hourly time-series products (2011). URL https://doi.org/10.5065/D6N877VB.

[13] Andresen, G. B., Söndergaard, A. A. & Greiner, M. Validation of Danish wind time series from a new global renewable energy atlas for energy system analysis. *Energy* **93**, 1074 – 1088 (2015). URL http://www.sciencedirect.com/science/article/pii/S0360544215012815.

[14] Schlachtberger, D. P., Brown, T., Schramm, S. & Greiner, M. The benefits of cooperation in a highly renewable European electricity network. *Energy* **134**, 469–481 (2017). URL http://www.sciencedirect.com/science/article/pii/S0360544217309969.
[15] Victoria, M. & Andresen, G. B. Using validated reanalysis data to investigate the impact of the PV system configurations at high penetration levels in european countries. *Progress in Photovoltaics: Research and Applications* **27**, 576–592. URL https://onlinelibrary.wiley.com/doi/full/10.1002/pip.3126.
[16] Victoria, M., Zhu, K., Brown, T., Andresen, G. B. & Greiner, M. The role of photovoltaics in a sustainable European energy system under variable CO_2 emissions targets, transmission capacities, and costs assumptions. *Progress in Photovoltaics: Research and Applications* URL https://onlinelibrary.wiley.com/doi/abs/10.1002/pip.3198.
[17] Corine land cover 2006. Tech. Rep., EEA (2014). URL https://www.eea.europa.eu/data-and-maps/data/clc-2006-raster-4.
[18] Natura 2000 data - the European network of protected sites. URL https://www.eea.europa.eu/data-and-maps/data/natura-8.
[19] Scholz, Y. *Renewable energy based electricity supply at low costs - development of the REMix model and application for Europe*. Ph.D. thesis, University of Stuttgart (2012). URL https://elib.uni-stuttgart.de/handle/11682/2032.
[20] Brown, T., Schlachtberger, D., Kies, A., Schramm, S. & Greiner, M. Synergies of sector coupling and transmission reinforcement in a cost-optimised, highly renewable European energy system. *Energy* **160**, 720–739 (2018). URL http://www.sciencedirect.com/science/article/pii/S036054421831288X.
[21] Technology Data for Generation of Electricity and District Heating, update November 2019. Tech. Rep., Danish Energy Agency and Energinet.dk (2019). URL https://ens.dk/en/our-services/projections-and-models/technology-data/technology-data-generation-electricity-and.
[22] Staffell, I. *et al.* The role of hydrogen and fuel cells in the global energy system. *Energy & Environmental Science* **12**, 463–491 (2019). URL https://pubs.rsc.org/en/content/articlelanding/2019/ee/c8ee01157e.
[23] Caglayan, D. *et al.* Technical Potential of Salt Caverns for Hydrogen Storage in Europe (2019). URL https://www.preprints.org/manuscript/201910.0187/v1.
[24] Brown, T., Schierhorn, P., Tröster, E. & Ackermann, T. Optimising the european transmission system for 77% renewable electricity by 2030. *IET Renewable Power Generation* **10**, 3–9.
[25] Ten-Year Network Development Plan 2016, ENTSOE. URL https://tyndp.entsoe.eu/maps-data/.
[26] Deliverable 3.1: Profile of heating and cooling demand in 2015. Data Annex. Heat Roadmap Europe. URL www.heatroadmap.eu.
[27] Population density by NUTS 3 region. URL https://data.europa.eu.
[28] Staffell, I., Brett, D., Brandon, N. & Hawkes, A. A review of domestic heat pumps. *Energy & Environmental Science* **5**, 9291–9306. URL https://pubs.rsc.org/en/content/articlelanding/2012/ee/c2ee22653g.
[29] Mapping and analyses of the current and future (2020 - 2030) heating/cooling fuel deployment (fossil/renewables). URL https://ec.europa.eu/energy/en/studies/mapping-and-analyses-current-and-future-2020-2030-heatingcooling-fuel-deployment.
[30] Euro Heat and Power. URL https://www.euroheat.org/knowledge-hub/country-profiles/.
[31] Zhu, K., Victoria, M., Andresen, G. B. & Greiner, M. Impact of climatic, technical and economic uncertainties on the optimal design of a coupled fossil-free electricity, heating and cooling system in Europe. *Applied Energy* **262**, 114500 (2020). URL http://www.sciencedirect.com/science/article/pii/S030626192030012X.
[32] Ruiz, P. *et al.* The JRC-EU-TIMES model. bioenergy potentials for EU and neighbouring countries. URL https://setis.ec.europa.eu/sites/default/files/reports/biomass_potentials_in_europe.pdf.
[33] ENSPRESO Biomass database, JRC. URL https://data.jrc.ec.europa.eu/dataset/74ed5a04-7d74-4807-9eab-b94774309d9f/resource/94aca7d6-89af-4969-a74c-2c7ab4376788.
[34] Wind energy database. URL https://www.thewindpower.net/.
[35] ODYSSEE database on energy effciency data & indicators. Tech. Rep. (2016). URL http://www.indicators.odyssee-mure.eu.
[36] Verkehrszahlung - Stundenwerte, Tech. Rep., Bundesanstalt für Straßenwesen.
[37] The circular economy – A powerful force for climate mitigation, Material Economics. Tech. Rep. (2018). URL https://www.sitra.fi/en/publications/circular-economy-powerful-force-climate-mitigation/.
[38] Vartiainen, E., Masson, G., Breyer, C., Moser, D. & Medina, E. R. Impact of weighted average cost of capital, capital expenditure, and other parameters on future utility-scale PV levelised cost of electricity. *Progress in Photovoltaics: Research and Applications* (2017). URL https://onlinelibrary.wiley.com/doi/abs/10.1002/pip.3189.
[39] Vartiainen, E., Masson, G. & Breyer, C. The true competitiveness of solar PV: a European case study. Tech. Rep., European Technology and Innovation Platform for Photovoltaics (ETIP) (2017). URL http://www.etip-pv.eu/fileadmin/Documents/ETIP_PV_Publications_2017-2018/LCOE_Report_March_2017.pdf.
[40] Lazard's Levelized Cost of Energy Analysis, version 13.0. URL https://www.lazard.com/media/451086/lazards-levelized-cost-of-energy-version-130-vf.pdf.
[41] Schröeder, A., Kunz, F., Meiss, F., Mendelevitch, R. & von Hirschhausen, C. Current and prospective costs of electricity generation until 2050, Data Documentation, DIW 68. Berlin: Deutsches Institut URL https://www.econstor.eu/handle/10419/80348.
[42] Hagspiel, S. *et al.* Cost-optimal power system extension under flow-based market coupling. *Energy* **66**, 654–666 (2014). URL http://www.sciencedirect.com/science/article/pii/S0360544214000322.
[43] Fasihi, M., Bogdanov, D. & Breyer, C. Long-Term Hydrocarbon Trade Options for the Maghreb Region and Europe—Renewable Energy Based Synthetic Fuels for a Net Zero Emissions World. *Sustainability* **9**, 306 (2017). URL https://www.mdpi.com/2071-1050/9/2/306.
[44] Schaber, K. *Integration of Variable Renewable Energies in the European power system: a model-based analysis of transmission grid extensions and energy sector coupling*. Ph.D. thesis, TU München (2013). URL https://d-nb.info/1058680781/34.
[45] Gerhardt, N., Scholz, A., Sandau, F. & H., H. Interaktion EE-Strom, Wärme und Verkehr. Tech. rep. Fraunhofer

IWES. URL http://www.energiesystemtechnik.iwes.fraunhofer.de/de/projekte/suche/2015/interaktion_strom_waerme_verkehr.html.
[46] Budischak, C. *et al.* Cost-minimized combinations of wind power, solar power and electrochemical storage, powering the grid up to 99.9% of the time. *Journal of Power Sources* **225**, 60–74 (2013). URL http://www.sciencedirect.com/science/article/pii/S0378775312014759.
[47] BP Statistical Review of World Energy. URL https://www.bp.com/content/dam/bp/business-sites/en/global/corporate/pdfs/energy-economics/statistical-review/bp-stats-review-2019-full-report.pdf.
[48] Development of the specific carbon dioxide emissions of the German electricity mix in the years 1990 - 2018, German Environment Agency. URL https://www.umweltbundesamt.de/sites/default/files/medien/1410/publikationen/2019-04-10_cc_10-2019_strommix_2019.pdf.
[49] Word Energy Outlook 2017, International Energy Agency. Tech. Rep.
[50] Zappa, W., Junginger, M. & van den Broek, M. Is a 100% renewable European power system feasible by 2050? *Applied Energy* **233-234**, 1027–1050 (2019). URL http://www.sciencedirect.com/science/article/pii/S0306261918312790.
[51] Wege Zu Einem Klimaneutralen Energiesystem - Anhang zur Studie. Tech. Rep. (2020). URL https://www.ise.fraunhofer.de/content/dam/ise/de/documents/publications/studies/Anhang-Studie-Wege-zu-einem-klimaneutralen-Energiesystem.pdf.
[52] Fell, H.-J., Breyer, C. & Metayer, M. The Projections for the Future and Quality in the Past of the World Energy Outlook for Solar PV and Other Renewable Energy Technologies. *31st European Photovoltaic Solar Energy Conference and Exhibition* 3220–3246 (2015). URL http://www.eupvsec-proceedings.com/proceedings?paper=35067.
[53] Creutzig, F. *et al.* The underestimated potential of solar energy to mitigate climate change. *Nature Energy* **2** (2017). URL https://www.nature.com/articles/nenergy2017140.
[54] Haegel, N. M. *et al.* Terawatt-scale photovoltaics: Transform global energy. *Science* **364**, 836–838 (2019). URL https://science.sciencemag.org/content/364/6443/836.
[55] Photovoltaics Report, Fraunhofer Institute for Solar Energy Systems, version November 2019. Tech. Rep. (2019). URL https://www.ise.fraunhofer.de/content/dam/ise/de/documents/publications/studies/Photovoltaics-Report.pdf.
[56] Child, M., Kemfert, C., Bogdanov, D. & Breyer, C. Flexible electricity generation, grid exchange and storage for the transition to a 100% renewable energy system in Europe. *Renewable Energy* **139**, 80–101 (2019). URL http://www.sciencedirect.com/science/article/pii/S0960148119302319.
[57] Bogdanov, D. *et al.* Radical transformation pathway towards sustainable electricity via evolutionary steps. *Nature Communications* **10**, 1–16 (2019). URL https://www.nature.com/articles/s41467-019-08855-1.
[58] In-depth analysis in support of the Comission Communication COM(2018) 773 A Clean Planet for all. A European long-term strategic vision for a prosperous, modern, competitive and climate neutral economy. Tech. Rep. (2018). URL https://ec.europa.eu/clima/news/commission-calls-climate-neutral-europe-2050_en.
[59] Technology pathways in decarbonisation scenarios. Tech. Rep. (2018). URL https://ec.europa.eu/energy/sites/ener/files/documents/2018_06_27_technology_pathways_-_finalreportmain2.pdf.
[60] Krey, V. *et al.* Looking under the hood: A comparison of techno-economic assumptions across national and global integrated assessment models. *Energy* **172**, 1254–1267 (2019). URL http://www.sciencedirect.com/science/article/pii/S0360544218325039.
[61] Egli, F., Steffen, B. & Schmidt, T. S. Bias in energy system models with uniform cost of capital assumption. *Nature Communications* **10**, 1–3 (2019). URL https://www.nature.com/articles/s41467-019-12468-z.
[62] Schyska, B. U. & Kies, A. How regional differences in cost of capital influence the optimal design of power systems. *Applied Energy* **262**, 114523 (2020). URL http://www.sciencedirect.com/science/article/pii/S0306261920300350.
[63] Evaluating our future. The crucial role of discount rates in European Commission energy system modelling, Ecofys. Tech. Rep. (2015). URL https://www.eurima.org/uploads/ModuleXtender/Publications/129/Ecofys_EvaluatingOurFuture-final20151019.pdf.
[64] Emmerling, J. *et al.* The role of the discount rate for emission pathways and negative emissions. *Environmental Research Letters* **14**, 104008 (2019). URL https://doi.org/10.1088%2F1748-9326%2Fab3cc9. Publisher: IOP Publishing.
[65] Renewable Energy and Jobs Annual Review 2019, IRENA. Tech. Rep. (2019). URL https://www.irena.org/publications/2019/Jun/Renewable-Energy-and-Jobs-Annual-Review-2019.
[66] Low carbon jobs: The evidence for net job creation from policy support for energy efficiency and renewable energy, UKERC. Tech. Rep. (2014). URL http://www.ukerc.ac.uk/programmes/technology-and-policy-assessment/low-carbon-jobs.html.
[67] Renewable Energy: a gender perspective. Tech. Rep. (2019). URL https://www.irena.org/-/media/Files/IRENA/Agency/Publication/2019/Jan/IRENA_Gender_perspective_2019.pdf.
